# Association between multimorbidity status and incident dementia: a prospective cohort study of 245,483 participants

**DOI:** 10.1038/s41398-022-02268-3

**Published:** 2022-12-07

**Authors:** He-Ying Hu, Ya-Ru Zhang, Qiaolifan Aerqin, Ya-Nan Ou, Zuo-Teng Wang, Wei Cheng, Jian-Feng Feng, Lan Tan, Jin-Tai Yu

**Affiliations:** 1 Department of Neurology, Qingdao Municipal Hospital, Qingdao University, Qingdao, China; 2 Department of Neurology and National Center for Neurological Disorders, Huashan Hospital, State Key Laboratory of Medical Neurobiology and MOE Frontiers Center for Brain Science, Shanghai Medical College, Fudan University, Shanghai, China; 3 The Institute of Science and Technology for Brain-inspired Intelligence, Fudan University, Shanghai, China

**Keywords:** Diseases, Psychiatric disorders

## Abstract

Multimorbidity (the presence of two or more long-term conditions [LTCs]) was suggested to exacerbate the neuronal injuries. The impact of multimorbidity on dementia has not been fully elucidated. We aimed to investigate the association between multimorbidity and dementia risk. We used the prospective data from 245,483 UK Biobank participants during a 9-year follow-up. Multimorbidity status was evaluated based on the LTC counts and multimorbidity patterns. Cox regression models adjusted for potential confounders were used to examine the associations of multimorbidity status with all-cause dementia (ACD), Alzheimer’s disease (AD) and vascular dementia (VD). Participants with multimorbidity at baseline had higher risks of ACD and VD, and the risks were elevated with the increase of LTC counts (ACD: hazard ratios [HR] = 1.15, 95% confidence intervals [CI] = 1.01–1.31 with 2 LTCs; HR = 1.18, CI = 1.01–1.39 with 3 LTCs; HR = 1.65, CI = 1.44–1.88 with ≥4 LTCs; VD: HR = 1. 66, CI = 1.24–2.21 with 2 LTCs; HR = 2.10, CI = 1.53–2.88 with 3 LTCs; HR = 3.17, CI = 2.43–4.13 with ≥4 LTCs). Participants with ≥4 LTCs also had a higher risk of AD (HR = 1.34, CI = 1.08–1.66]. Participants with the cardio-cerebrovascular/respiratory/metabolic/musculoskeletal/depressive multimorbidity were 1.46, 1.28, and 2.50 times more likely to develop ACD (HR = 1.46, 95% CI = 1.28–1.67), AD (HR = 1.28, CI = 1.04–1.58), and VD (HR = 2.50, CI = 1.90–3.27), respectively. Those with tumor/genitourinary/digestive disorders had a 11% higher hazard of ACD (HR = 1.11, CI = 1.00–1.24) and a 73% elevated risk of VD (HR = 1.73, CI = 1.37–2.18). The prevention of LTC accumulation and the identification of specific multimorbidity patterns might be beneficial to the prevention of dementia and its subtypes, AD as well as VD.

## Introduction

Dementia has gradually become one of the leading public health concerns resulting from worldwide population aging [1]. As a major cause of disability, dependency, and mortality, dementia poses a heavy burden on patients, families and societies [2, 3]. Dementia is a highly heterogeneous disease composed of various subtypes, with the most common subtype of Alzheimer’s disease (AD) (60–70%) followed by vascular dementia (VD) (25%) [4, 5]. The incidence of dementia is affected by diverse factors. Long-term conditions (LTCs) such as hypertension, diabetes, atrial fibrillation (AF), and depression [6–9] were found to be related to a higher risk of dementia. However, the relationship between multiple comorbid LTCs (multimorbidity) and dementia is still unclear.

Several studies indicated that multimorbidity (the presence of at least two LTCs) was associated with accelerated neuronal injury and severe dementia pathologies [10]. Several longitudinal studies further showed that patients with multimorbidity were at an increased risk of cognitive decline or dementia [11–13]. However, whether those with an increased number of LTCs experienced a higher cumulative risk was not well studied. Moreover, due to the overlapping risk factors or shared pathophysiological mechanisms, some conditions tended to coexist in the same individual, leading to multimorbidity patterns featuring LTCs that systematically cluster together rather than by chance [14]. To date, there is scarce evidence on how LTCs co-occurr in single individuals and how clusters of diseases are differentially linked to dementia incidence. A recent study identified the clusters of LTCs in the elderly and explored the associations of different multimorbidity patterns with the risk of all-cause dementia (ACD), but the sample size (*n* = 2478) was relatively small. Besides, the studies exploring the associations between multimorbidity and dementia subtypes (e.g., AD and VD) are rare [15].

Previous evidence suggested that the incidences of adverse health outcomes (e.g., obesity, diabetes, and cardiovascular disease) varied by sex [16]. Whether the impact of health conditions on dementia risk differed between males and females has not been examined. Besides, the impacts of age and the *apolipoprotein E4* (*APOE4*) carrier status on dementia risk was well studied. Whether age or *APOE4* status could modify the impact of multimorbidity on the development of dementia was unclear.

Therefore, using the large-scale data from 245,483 older adults from the UK Biobank (UKB), we identified the clusters of LTCs in patterns of multimorbidity and performed a prospective cohort study to examine (1) the impact of LTC counts and specific multimorbidity patterns on the risk of dementia (including ACD, AD, and VD); as well as (2) the effect modification by sex, age, or *APOE4* status.

## Methods

### Study design and participants

This is a large-scale prospective cohort study of participants enrolled from the UKB, which recruited 502,464 participants aged 37–73 years between 2006 and 2010. The participants attended 1 of 22 assessment centers across the United Kingdom, and completed the touchscreen and nurse-led questionnaires. Data from the clinic, as well as data on genetic and risk factors were obtained at baseline. Since recruitment, the participants have been followed for clinical outcomes (including dementia) via hospital inpatient records, death certificates and primary care records. The UKB study received approval from the National Health Service (NHS) NorthWestMulticenter Research Ethics Committee. All participants provided written informed consent. This study included participants with hospital inpatient diagnoses (International Classification of Diseases, Tenth Revision [ICD-10] data). Individuals younger than 55 years at baseline were excluded since they had a low risk of incident dementia which might bias the conclusion. What’s more, we excluded participants with prevalent dementia at baseline and those who lost follow-up, leaving 245,483 participants included.

### Assessment of LTCs

The health conditions of participants were assessed at the time of study recruitment. Previous studies have reported the definition of chronic disease and identified the codes as chronic based on the ICD-10 data. These codes were finally grouped into 59 LTC categories according to a clinically driven methodology [15, 17]. The description of the ICD-10 codes included in each LTC category in the UKB was shown in Supplementary Table 1.

### Dementia diagnoses

In this study, ACD, AD, and VD cases were ascertained when the algorithmically-defined dementia occurred (Fields 42,018–42,025); the first-occurrence data reported dementia onsets within nervous system disorders (Fields 131,036–131,037) or mental and behavioral disorders (Fields 130,836–130,843); the death register data documented dementia as the primary or secondary causes of death (Fields 40,001–40,002); the hospital inpatient data recorded dementia diagnoses at admission (Fields 41,270–41,271, 41,280–41,281); and the primary care data reported dementia-related events (Field 42,040). Dementia was diagnosed and classified according to the ICD codes (ICD-9 and ICD-10) and Read codes (version 2 [Read v2] and version 3 [Read v3]). Apart from AD and VD, ACD covered other dementia classifications, including frontotemporal dementia (FTD), dementia with Lewy bodies (DLB), Parkinson’s disease dementia (PDD), and dementia in other neurodegenerative or specified diseases (e.g., corticobasal degeneration, Huntington’s disease). Incident dementia was defined as dementia diagnosed after the date of baseline assessment (Field 53). Follow-up visits began on date of attending assessment center. The participants were followed up to the date of the earliest incident dementia diagnosis, the last data collection date by general practitioners, the date of the last hospital inpatient admission, or the date of death, whichever happened first.

### Statistical analysis

To avoid statistical noise, we excluded LTCs with a prevalence of <1%, leaving 29 individual LTCs included in the following analyses. The prevalences of 59 LTCs at baseline were shown in Supplementary Table 2. Based on LTC counts, multimorbidity was classified into 0 LTC (healthy), 1 LTC, 2 LTCs, 3 LTCs, 4 or more LTCs. Next, a fuzzy c-means cluster analysis algorithm was used to identify the multimorbidity patterns. Individuals with at least one LTC (*n* = 137,823 [56.14%]) were divided into three clusters based on their underlying combinations of chronic diseases at baseline. To be specific, a probability of cluster membership for all individuals was assigned within each cluster, whereas participants were finally allocated a single cluster based on their highest membership probability. To characterize the multimorbidity patterns, disease exclusivity was calculated by dividing the number of participants with the LTC included in a cluster by the total number of participants with the LTC. Besides, the observed/expected ratio was equal to the prevalence of a given LTC within a cluster divided by its prevalence in the total population. The details of LTCs in each cluster were described in Supplementary Table 3. Diseases with both exclusivity ≥25% and observed/expected ratio ≥2 were qualified for characterizing a given cluster (see Supplementary Table 4). The above criteria were used to name the multimorbidity patterns based on the LTCs which characterized them. Details on the methodology were reported elsewhere [15]. Baseline sociodemographic and multimorbidity characteristics of the participants stratified by dementia status (n dementia and no incident dementia) were summarized. Intergroup comparisons were performed using the *t*-test for normally distributed data and the chi-square test for frequencies.

Before our primary analyses, we compared the risk of dementia between participants with individual LTCs and healthy controls using Cox proportional hazard regression models. In the primary analyses, survival plots showed the cumulative dementia incidences within groups of different multimorbidity statuses (including LTC count categories and multimorbidity patterns). Cox proportional hazard regression models were used to examine the association between multimorbidity status at baseline and dementia risk, with healthy participants as reference and the duration of follow-up as the timescale. Participants who died, withdrew from the study before the end of follow-up, or had not developed dementia by the end of follow-up were censored (i.e., non-cases). Cox regression models were adjusted for age at baseline (continuous), sex (female/male), education (higher [with college/university degree or other professional qualification]/ lower), body mass index (BMI) (continuous), physical activity (composed of walking, moderate activity and vigorous activity) (continuous), smoking status (never/ previous/ current), and *APOE4* carrier status (carrier/ non-carrier status as defined by genetic information). Hazard ratios (HRs) with 95% confidence intervals (CIs) were reported. We performed separate analyses for ACD, AD, and VD. To investigate whether sex, age, and *APOE4* status modified the effects of multimorbidity status on dementia, the subgroup analyses stratified by sex, age (the mid-aged [<65 years old] and the old-aged [≥65 years old]), and *APOE4* status were conducted. A *P*-value <0.05 was considered to be statistically significant. R version 4.0.5 [18] and SPSS 25.0 were used for statistical analyses and figure preparation.

## Results

### Participant characteristics

**Fig. 1:**
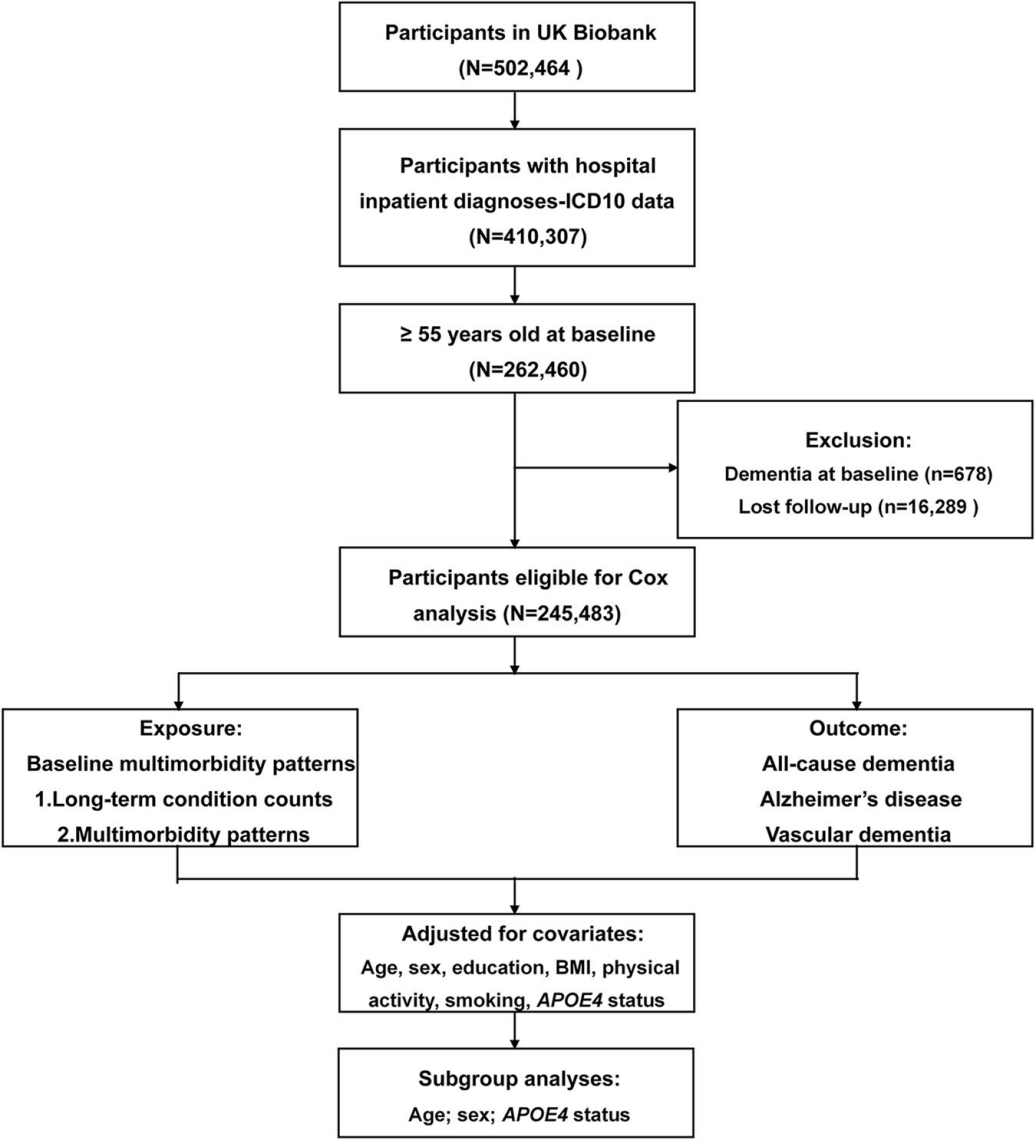
Flowchart of participant selection. ICD-10 International Classification of Diseases, Tenth Revision, BMI body mass index, *APOE4 apolipoprotein E4*.

At baseline, a total of 245,483 participants in UKB were included in this study. The flowchart of participant selection was shown in Fig. 1. The mean age of the population was 62.32 years (standard deviation [SD] 4.08); 53.16% were women; and 57.48% were higher educated. A total of 34,741 (14.15%) participants had 2 LTCs; 20,299 (8.27%) had 3; and 28,630 (11.66%) had ≥4. Besides, the following three multimorbidity patterns were identified: obesity accompanied with other disorders (Pattern A, *n* = 46,172 [18.81%]), cardio-cerebrovascular/respiratory/metabolic/musculoskeletal/depressive disorders (Pattern B, *n* = 24,504 [9.98%]), and tumor/genitourinary/digestive disorders (Pattern C, *n* = 67,147 [27.35%]) (Table 1). Supplementary Table 5 presented a list of LTCs included in each pattern.

**Table 1:** Baseline characteristics of UKB participants stratified by incident dementia status.

Variables	All participants (*n* = 245,483)	No incident dementia (*n* = 240,360)	Incident ACD (*n* = 5123)	*P-*value	Incident AD (*n* = 2228)	*P-*value	Incident VD (*n* = 1234)	*P*-value
Age, mean (SD), y	62.32 (4.08)	62.26 (4.07)	65.07 (3.50)	<0.001^b^	65.21 (3.35)	<0.001^b^	65.26 (3.43)	<0.001^b^
Female, *n* (%)	130,497 (53.16)	128,127 (53.31)	2370 (46.26)	<0.001^c^	1144 (51.35)	0.065^c^	500 (40.52)	<0.001^c^
Higher education ^a^, where reported, *n* (%)	103,005 (57.48)	101,364 (57.55)	1641 (53.42)	<0.001^c^	683 (51.55)	<0.001^c^	345 (50.51)	<0.001^c^
BMI, where reported, mean (SD), kg/m^2^	27.78 (4.75)	27.78 (4.74)	27.89 (5.00)	0.120^b^	27.41 (4.75)	<0.001^b^	28.66 (5.14)	<0.001^b^
Physical activity, where reported, mean (SD), MET-min/week	2684.33 (2718.95)	2685.45 (2715.30)	2627.95 (2897.18)	0.230^b^	2870.32 (3008.37)	0.014^b^	2463.86 (2744.87)	0.015^b^
Smoking status, where reported, *n* (%)				<0.001^c^		0.018^c^		<0.001^c^
Current	22,679	22,142	537	—	202	—	147	—
Previous	99,628	97,386	2242	—	961	—	569	—
Never	121,536	119,256	2280	—	1036	—	498	—
*APOE4* carriers, where reported, *n* (%)	6,1372 (28.49)	59,015 (27.96)	2357 (54.23)	<0.001^c^	1207 (63.06)	<0.001^c^	526 (50.82)	<0.001^c^
LTC counts, *n* (%)				<0.001^c^		<0.001^c^		<0.001^c^
0	107,660 (43.86)	105,910 (44.06)	1750 (34.16)	—	827 (37.12)	—	314 (25.45)	—
1	54,153 (22.06)	53,176 (22.12)	977 (19.07)	—	433 (19.43)	—	219 (17.75)	—
2	34,741 (14.15)	33,968 (14.13)	773 (15.09)	—	350 (15.71)	—	181 (14.67)	—
3	20,299 (8.27)	19,771 (8.23)	528 (10.31)	—	211 (9.47)	—	141 (11.43)	—
4 or more	28,630 (11.66)	27,535 (11.46)	1095 (21.37)	—	407 (18.27)	—	379 (30.71)	—
Multimorbidity patterns, *n* (%)				<0.001^c^		<0.001^c^		<0.001^c^
Healthy	107,660 (43.86)	105,910 (44.06)	1750 (34.16)	—	827 (37.12)	—	314 (25.45)	—
Pattern A (obesity accompanied with other disorders)	46,172 (18.81)	45,205 (18.81)	967 (18.88)	—	359 (16.11)	—	284 (23.01)	—
Pattern B (cardio-cerebrovascular/respiratory/metabolic/musculoskeletal/depressive disorders)	24,504 (9.98)	23,545 (9.80)	959 (18.72)	—	385 (17.28)	—	304 (24.64)	—
Pattern C (tumor/genitourinary/digestive disorders)	67,147 (27.35)	65,700 (27.33)	1447 (28.25)	—	657 (29.49)	—	332 (26.90)	—

Data presented as mean (SD) for continuous variables and number (%) for categorical variables. Among incident dementia cases, ACD, AD, and VD were treated as separate outcomes.

*UKB* UK Biobank, *ACD* all-cause dementia, *AD* Alzheimer’s disease, *VD* vascular dementia, *SD* standard deviation, *BMI* body mass index, *MET* metabolic equivalent, *APOE4* apolipoprotein E4, *LTC* long-term condition.

^a^Higher education refers to “with college/university degree or other professional qualification”.

^b^Comparisons between incident dementia group and no incident dementia group were performed via the *t*-test ^b^ or

^c^Chi-square test.

During a median follow-up of 9.26 years (interquartile range [IQR] 7.15–10.78), 5,123 participants (2.09%) developed dementia; 2228 participants (0.91%) developed AD; and 1234 participants (0.50%) developed VD. Baseline characteristics of participants stratified by incident dementia status were shown in Table 1. Demented cases were older, more frequently males, less educated, more likely to smoke, more likely to be *APOE4* carriers, and more likely to be multimorbid (*P* < 0.001 for all). Participant characteristics across eight categories according to the multimorbidity status were shown in Supplementary Table 6.

### Associations of the individual LTCs at baseline with the risk of dementia

**Fig. 2:**
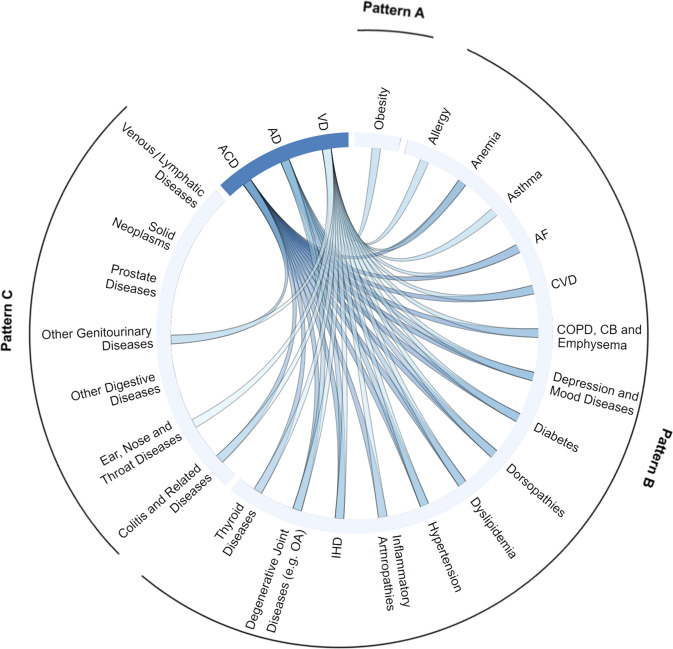
Circos-plot to visualize relationships between LTCs in different multimorbidity patterns and dementia risk. After adjusting for age, sex, education, BMI, physical activity, smoking, and *APOE4* status, 18 of the 29 individual LTCs at baseline were associated with an increased risk of ACD. Seven LTCs were associated with a higher incidence of AD, and 24 LTCs were associated with a higher incidence of VD. Analyses were performed by Cox proportional hazard regression. LTC long-term condition, ACD all-cause dementia, AD Alzheimer’s disease, VD vascular dementia, IHD ischemic heart disease, OA osteoarthritis, AF atrial fibrillation, CVD cerebrovascular disease, COPD chronic obstructive pulmonary disease, CB chronic bronchitis, BMI body mass index, *APOE4 apolipoprotein E4*.

After adjusting for age, sex, education, BMI, physical activity, smoking, and *APOE4* status, 18 of the 29 individual LTCs at baseline were significantly associated with an increased risk of ACD. As for the subtypes, 7 LTCs were associated with a higher incidence of AD, and 24 LTCs were associated with a higher incidence of VD. HRs and 95% CIs for ACD, AD, and VD according to the individual LTCs at baseline were reported in Supplementary Table 7. Figure 2 summarized the relationships between individual LTCs in different multimorbidity patterns and ACD, AD or VD risks. Results of subgroup analyses stratified by sex, age, and *APOE4* status were reported in Supplementary Tables 8–13.

### Associations between LTC counts and risks of ACD, AD, and VD

**Fig. 3:**
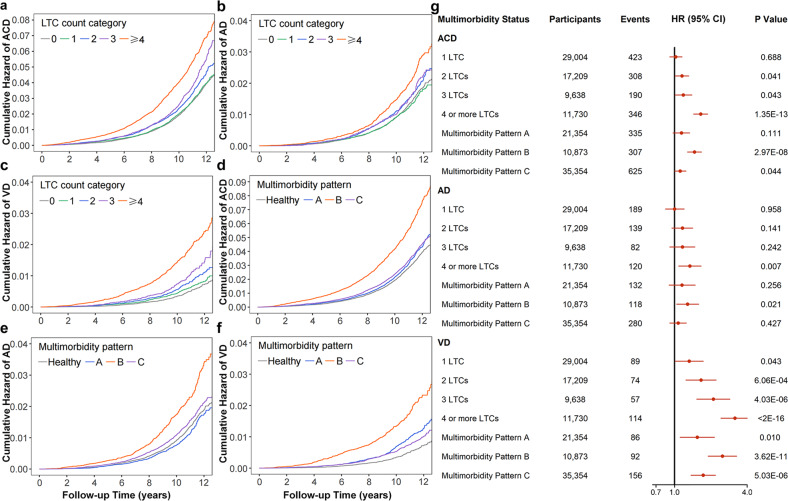
Associations between multimorbidity status and risks of ACD, AD, and VD. Compared with healthy controls, participants with ≥ 2 LTCs had steeper gradients in the cumulative incidence curves for ACD (**a**), AD (**b**), and VD (**c**). Participants with any multimorbidity pattern had steeper gradients in the cumulative incidence curves for ACD (**d**) and VD (**f**). Participants with multimorbidity patterns B and C had a higher incidence of AD (**e**). After adjusting for age, sex, education, BMI, physical activity, smoking, and *APOE4* status, participants with ≥2 LTCs at baseline had higher risks of ACD and VD, with the increase of LTC counts leading to cumulative hazards. Participants with ≥4 LTCs had a higher risk of AD (**g**). Besides, multimorbidity pattern B and pattern C were associated with a higher risk of ACD. Only pattern B had a harmful impact on AD, while all of the three patterns had harmful impacts on VD (**g**). *P* < 0.05 for all. *P*-values were computed by Cox proportional hazard regression. LTC long-term condition, ACD all-cause dementia, AD Alzheimer’s disease, VD vascular dementia, HR hazard ratios, CI confidence interval, BMI body mass index, *APOE4 apolipoprotein E4*.

During the follow-up period, the numbers of dementia cases within four LTC count categories were reported in Supplementary Table 6. Compared with healthy controls, participants with two or more LTCs had steeper gradients in the cumulative incidence curves for ACD, AD, and VD. Participants with ≥4 LTCs had the highest incidences of ACD, AD, and VD throughout the follow-up period (Fig. 3a–c).

Results of the adjusted Cox regression model (by age, sex, education, BMI, physical activity, smoking, and *APOE4* status) were shown in Table 2 and Fig. 3g. A dose-response relationship between LTC counts at baseline and the risk of ACD was observed. Concretely, no significant difference was found in the ACD risk between participants with 1 LTC and healthy participants (HR = 1.02 [95% CI: 0.91–1.15] for 1 LTC, *P* = 0.688). Participants with 2 LTCs and those with 3 LTCs at baseline were 1.15 times (95% CI: 1.01–1.31; *P* = 0.041) and 1.18 times (95% CI: 1.01–1.39; *P* = 0.043) more likely to develop ACD, respectively. Participants with ≥4 LTCs had a more than 1.6-fold higher risk of ACD (HR = 1.65 [95% CI: 1.44–1.88], *P* < 0.001). In terms of separate outcomes, participants with ≥4 LTCs had a higher risk of AD (HR = 1.34 [95% CI: 1.08–1.66], *P* = 0.007). The risks of VD were elevated with the increase of LTC counts, and the results were as follows: HR = 1.32 (95% CI: 1.01–1.73) for 1 LTC, *P* = 0.043; HR = 1.66 (95% CI: 1.24–2.21) for 2 LTCs, *P* < 0.001; HR = 2.10 (95% CI: 1.53–2.88) for 3 LTCs, *P* < 0.001; HR = 3.17 (95% CI: 2.43–4.13) for ≥4 LTCs, *P* < 0.001.

**Table 2:** Associations between multimorbidity status and incident dementia in UKB.

Multimorbidity status	ACD				AD				VD			
	Participants	Events	HR (95% CI)	*P*-value	Participants	Events	HR (95% CI)	*P-*value	Participants	Events	HR (95% CI)	*P*-value
Counts												
0 LTC	61,629	804	Reference	—	61,629	368	Reference	—	61,629	130	Reference	—
1 LTC	29,004	423	1.02 (0.91–1.15)	0.688	29,004	189	1.00 (0.84–1.2)	0.958	29,004	89	1.32 (1.01–1.73)	**0.043**
2 LTCs	17,209	308	1.15 (1.01–1.31)	**0.041**	17,209	139	1.16 (0.95–1.41)	0.141	17,209	74	1.66 (1.24–2.21)	**6.06E-04**
3 LTCs	9638	190	1.18 (1.01–1.39)	**0.043**	9638	82	1.16 (0.91–1.47)	0.242	9638	57	2.10 (1.53–2.88)	**4.03E-06**
4 or more LTCs	11,730	346	1.65 (1.44–1.88)	**1.35E-13**	11,730	120	1.34 (1.08–1.66)	**0.007**	11,730	114	3.17 (2.43–4.13)	**<2E-16**
Patterns												
Healthy	61,629	804	Reference	—	61,629	368	Reference	—	61,629	130	Reference	—
Multimorbidity Pattern A	21,354	335	1.14 (0.97–1.33)	0.111	21,354	132	1.15 (0.9–1.48)	0.256	21,354	86	1.54 (1.11–2.14)	**0.010**
Multimorbidity Pattern B	10,873	307	1.46 (1.28–1.67)	**2.97E-08**	10,873	118	1.28 (1.04–1.58)	**0.021**	10,873	92	2.50 (1.90–3.27)	**3.62E-11**
Multimorbidity Pattern C	35,354	625	1.11 (1.00–1.24)	**0.044**	35,354	280	1.07 (0.91–1.25)	0.427	35,354	156	1.73 (1.37–2.18)	**5.03E-06**

HRs and 95% CIs for dementia according to multimorbidity status were calculated using Cox proportional hazard regression with healthy participants as reference after adjusting for age, sex, education, BMI, physical activity, smoking, and *APOE4* status.

*UKB* UK Biobank, *ACD* all-cause dementia, *AD* Alzheimer’s disease, *VD* vascular dementia, *LTC* long-term condition, *BMI* body mass index, *APOE4*, *apolipoprotein E4*.

Bold values indicates statistically significant p values (*P* < 0.05).

### Associations between multimorbidity patterns and risks of ACD, AD, and VD

The numbers of dementia cases within different multimorbidity pattern groups were shown in Supplementary Table 6. Compared with healthy population, participants with multimorbidity patterns A, B, and C had steeper gradients in the cumulative incidence curves for ACD and VD. Participants with multimorbidity patterns B and C had a higher incidence of AD. It is worth noting that participants with multimorbidity pattern B had the highest incidences of ACD, AD, and VD throughout the follow-up period (Fig. 3d–f).

Results of the adjusted Cox regression model (by age, sex, education, BMI, physical activity, smoking, and *APOE4* status) were reported in Table 2 and Fig. 3g. Compared with healthy controls, participants with multimorbidity pattern B and those with multimorbidity pattern C at baseline showed a 46% (HR = 1.46 [95% CI: 1.28–1.67], *P* < 0.001) higher risk and a 11% (HR = 1.11 [95% CI: 1.00–1.24], *P* = 0.044) higher risk of ACD, respectively. As for separate outcomes, participants with pattern B experienced a higher hazard of AD (HR = 1.28 [95% CI: 1.04–1.58], *P* = 0.021). In terms of VD, significantly harmful effects were shown within all of the three multimorbidity patterns (pattern A, HR = 1.54 [95% CI: 1.11–2.14]; pattern B, HR = 2.50 [95% CI: 1.90–3.27]; pattern C, HR = 1.73 [95% CI: 1.37–2.18]; *P* < 0.05 for all).

### Subgroup analyses for the associations by sex, age, and *APOE4* status

**Fig. 4:**
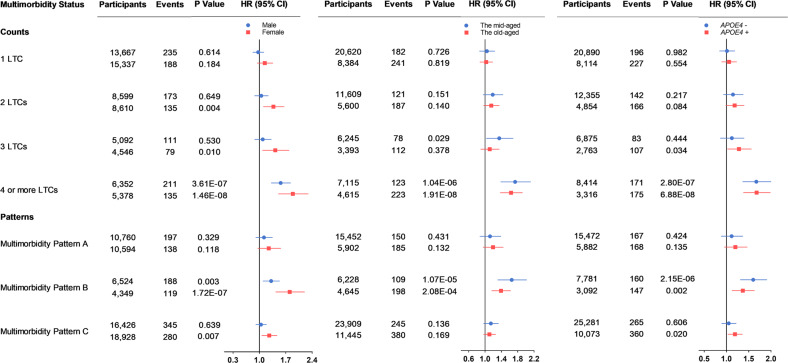
Subgroup analyses for the association between multimorbidity status and ACD by sex, age, and *APOE4* status. After adjusting for age, education, BMI, physical activity, smoking, and sex or *APOE4* status, females with ≥2 LTCs and pattern B or C at baseline had a higher hazard of ACD, while males with ≥4 LTCs and pattern B had a higher hazard. Besides, the associations of multimorbidity with ACD did not obviously differ by age and *APOE4* status. *P* < 0.05 for all. *P-*values were computed by Cox proportional hazard regression. ACD all-cause dementia, BMI body mass index, *APOE4 apolipoprotein E4*, LTC long-term condition, HR hazard ratios, CI confidence interval.

When analyses were stratified by sex, the associations of multimorbidity with the risks of ACD and AD was more pronounced in females, but not the risk of VD (see Supplementary Table 14). The associations of multimorbidity with dementia did not obviously differ by age and *APOE4* status (see Supplementary Tables 15–16). Results of subgroup analyses for ACD stratified by sex, age, and *APOE4* status were shown in Fig. 4.

## Discussion

Leveraging data of 245,483 individuals from the UKB, this study investigated the association between multimorbidity status and dementia over the 9-year follow-up. Our results showed that older adults with multimorbidity had a higher risk of dementia, with the increase of LTC counts at baseline leading to cumulative hazards of ACD and VD. Another major finding was that older adults with the cardio-cerebrovascular/respiratory/metabolic/musculoskeletal/depressive multimorbidity experienced the highest hazards of ACD, AD, and VD, followed by those with tumor/genitourinary/digestive disorders. The identification of older adults with multimorbidity might be beneficial in preventing dementia.

The major findings were mostly consistent with previous evidence. Previous observational studies have suggested that multimorbidity was associated with the occurrence of cognitive impairment or dementia, but the cross-sectional designs limited the exploration for causality [19–21]. Recently, Ben et al. conducted a prospective study of 10,095 adults and found that multimorbidity, particularly severe multimorbidity (≥3 LTCs), was associated with dementia risk [13]. Another cohort study of 2478 elders (aged ≥60 years) by Grande et al. found the neuropsychiatric/cardiovascular/sensory impairment/cancer multimorbidity to be associated with higher dementia incidence [15]. For the comprehensive evaluation of multimorbidity status among participants, our study not only used quantitative approaches (reflected in LTC counts) but also captured the clustering of LTCs in patterns of multimorbidity. To the best of our knowledge, this cohort study is the first to follow a large sample of more than 240,000 participants aged ≥50 years and examined the impact of multimorbidity status on dementia as well as separate outcomes (AD and VD).

Compared with healthy controls, a significantly increased dementia risk was found in participants with ≥2 LTCs rather than those with only one LTC, indicating that the coexistence of two or more LTCs might put patients at a higher risk of dementia. Compared to an individual LTC, multimorbidity could reflect the deterioration of health condition as a whole. Previous evidence suggested that the longstanding coexistence of diseases was associated with neurodegenerative biomarkers and it aggravated brain pathologies before the symptom onset of dementia [10, 22]. Importantly, we found increased LTC counts at baseline to be associated with an elevated risk of dementia. A possible explanation was that participants with worse health condition were more likely to have a systemic and chronic inflammatory state, and neuroinflammation was suggested to be a driving force behind the development of dementia [15, 23]. Besides, increased counts of LTCs were associated with a greater possibility of polypharmacy and heavier treatment burden, which might affect brain and cause neural injuries [24–26]. Moreover, a potential reason for the positive association between LTC counts and dementia risk was aging, as participants with a higher count of LTCs were more likely to be older.

Obesity accompanied with other disorders (pattern A) had a robust association with VD risk. Obesity is a well-known risk factor for cerebrovascular diseases (CVD). Obesity might reduce blood supply to the brain [27]. Fat cells would secrete proinflammatory cytokines and damage the white matter in brain, leading to cognitive deficits [28]. Notably, the cumulative survival plot showed a trend for the association between this cluster and a higher ACD incidence, but the effect size did not reach a statistically significant level in the adjusted Cox model. Although obesity is a well-known risk factor for dementia [29], the association between pattern A and ACD risk was non-significant. One explanation was that we did not investigate the effects of individual diseases on dementia risk but investigated the dementia risk conveyed by belonging to a cluster of individuals displaying a given disease combination. The detrimental impact of obesity might be diluted by other LTCs of this cluster. Another explanation was that obesity was defined as a BMI of ≥30 Kg/m^2^ in this study, but the potential non-linear association of BMI with ACD risk was not examined here [29].

In this study, older adults with the cardio-cerebrovascular/respiratory/metabolic/musculoskeletal/depressive multimorbidity pattern were found to experience higher risks of ACD, AD, and VD. Previous evidence suggested that people with circulatory or respiratory systemic diseases were at higher risks of poor prognoses including cognitive decline and dementia [30, 31]. Chronic cerebral hypoperfusion or hypoxia resulting from heart or lung diseases has been suggested to cause blood-brain barrier dysfunction and accelerate AD-related pathologies including amyloid-β (Aβ) plaques and neurofibrillary tangles [32, 33]. The mechanisms underlying the heart-brain connection also included hypercoagulable state, brain infarcts, cardiogenic brain embolism, and chronic inflammation [34, 35]. Hypertension, hyperlipidemia and diabetes have also been identified as independent predictors of both AD and VD [7, 8, 36]. Although the association of chronic arthropathies with dementia was under debate, the inflammatory cytokines released from osteoarthritis (OA) joints were found to reach the brain tissue, leading to cerebrovascular alteration, neuroinflammation and accelerated deposition of Aβ [37–39]. Although depression could be a prodromal manifestation of dementia, longitudinal studies have identified depression as an important risk factor for AD, and the potential mechanisms included an imbalance of stress hormones, the reduced hippocampal volume and increased systemic inflammation [40, 41]. Besides, LTCs of this cluster tended to co-occur partly because they shared some similar pathophysiological mechanisms or risk factors with each other, and the factors also had an impact on brain dysfunction. For instance, these LTCs could co-occur as an expression of biological degenerative changes occurring with age. Moreover, individuals in this cluster were more likely to be older and vulnerable. Low-grade chronic proinflammatory state was very frequent in older adults with multimorbidity, which could in turn increase the risk of the above-mentioned LTCs [15]. Meanwhile, neuroinflammation was found to be an epiphenomenon of neuronal loss and a driving force in the development of dementia [42]. Finally, certain therapeutic regimens might have adverse side effects on brain function. For example, the long-term insulinotropic oral antidiabetics could cause hypoglycemia and cognitive deficits in older adults; the treatment with anticholinergic drugs for respiratory or depressive disorders could also affect cognition [43, 44]. Importantly, patients in this cluster were more likely to have complex therapy-management, where polypharmacy could cause potential drug-drug interactions as well as enlarge side effects of neural injuries [26].

Genitourinary/digestive disorders and tumor clustered together possibly because that they shared overlapped risk factors including obesity, unhealthy lifestyle or diet and bacterial infection. The mechanisms underlying relationships between this cluster and ACD or VD were unclear. Although evidence relating genitourinary disorders and dementia was scarce, previous studies showed that patients with prostatic diseases and erectile dysfunction were more likely to be obese [45]. Recently, intestinal microbial imbalance, irritable bowel syndrome, vitamin B12, or folate deficiency caused by absorption dysfunction, and the long-term use of proton pump inhibitors were suggested to increase dementia risk in older adults [46–48]. The relationship between tumor and dementia risk was complex and could be influenced by tumor type, tumor location and therapeutic regimens [49–52]. Notably, genitourinary and digestive disorders were closely related to the risk of solid neoplasms in the corresponding organs. Tumor shared common risk factors or mechanisms with dementia, including but not limited to smoking, diabetes, obesity, chronic inflammation, and aging [52]. Certain cancer chemotherapy and adjuvant therapy agents could have neurotoxic effects [53]. For example, the androgen-deprivation therapy for prostate cancer and chemotherapy for colorectal cancer were associated with increased risks of drug-induced cognitive decline and dementia [54–58]. Furthermore, patients in this cluster were more likely to suffer from malnutrition and even cachexia due to the dysfunction of digestive system or invasion of tumor cells. Finally, this cluster grouped a higher percentage of females and older or low-educated participants who were more likely to be with lower levels of cognitive performance [15].

The associations of multimorbidity with ACD and AD were more evident in females. Previous studies have identified the sex differences in the prevalences of LTCs (e.g., obesity, diabetes, cardiovascular disease, and stroke) among population [16, 59]. Importantly, the impact of individual LTCs on dementia risk could vary by sex [60, 61]. Besides, sex could modulate the susceptibility to dementia [16]. In our study, the sex difference in the association were mainly observed for ACD and AD. Correspondingly, epidemiologic studies found females to be at greater risks of developing ACD and AD dementia, while males at a greater risk of developing VD [62]. Furthermore, the structure and function of brain were suggested to differ between males and females throughout the aging process, which might lead to the sex difference in the multimorbidity-dementia relationship [63].

This study has several strengths. We had a large sample size and long follow-up time. In addition, we analyzed not only the number but also the patterns of diseases, therefore the multimorbidity-dementia relationship was more convincing. Besides, the methodology used for pattern identification helped us describe the overall health status of a specific population, providing useful clinical information. This study also has some limitations. Firstly, our data were insufficient to study the role played by drug interaction and the dynamic changes in multimorbidity status during follow-up. Secondly, the cluster analysis was a fuzzy algorithm, and the identification of multimorbidity patterns was hardly comparable with other studies because of inconsistent population selected. Anyway, identifying the specific population who were at a high risk of dementia was of great value. Thirdly, the UKB is a population-based study, lacking biological data for the insight into mechanisms underlying the associations that were observed.

## Conclusion

Multimorbidity status (including LTC counts and multimorbidity patterns) was shown to be associated with the risks of dementia and its subtypes (AD and VD). The LTC types as well as LTC counts could help to understand the relationship between multimorbidity and dementia better. The cardio-cerebrovascular/respiratory/metabolic/musculoskeletal/depressive multimorbidity had associations with ACD, AD, and VD, while the tumor/genitourinary/digestive disorders multimorbidity only had associations with ACD and VD. The timely identification of multimorbidity (especially specific patterns) and the prevention of disease accumulation might be important to the primary prevention of dementia and the optimal allocation of health care resources among older adults.

## Supplementary information

Supplemental files

## Data Availability

The datasets supporting the conclusions of this study are available in the UK Biobank (http://www.ukbiobank.ac.uk/) with an approved protocol. External investigators can request the data and approval of use on application to the UK Biobank.
