# Supplementary material for: Association between multimorbidity status and incident dementia: a prospective cohort study of 245,483 participants
Source: Transl Psychiatry. 2022 Dec 7;12:505. doi: 10.1038/s41398-022-02268-3 (PMC9729184; doi:10.1038/s41398-022-02268-3)
Supplement: Supplementary file 1 — Supplemental files [file 41398_2022_2268_MOESM1_ESM.docx]

**Contents**

| **TITLE** | **PAGE** |
| --- | --- |
| **Supplementary Table 1: Description of ICD-10 codes included in each LTC category in the UKB** | 2 |
| **Supplementary Table 2: The prevalences of LTCs at baseline in the study population** | 24 |
| **Supplementary Table 3: Information of LTCs at baseline in each cluster** | 27 |
| **Supplementary Table 4: LTCs that have the potential to characterize each cluster** | 30 |
| **Supplementary Table 5: The list of LTCs included in each pattern** | 32 |
| **Supplementary Table 6: Participant characteristics across eight categories according to the multimorbidity status** | 33 |
| **Supplementary Table 7: Risks of dementia in participants with individual LTCs at baseline** | 35 |
| **Supplementary Table 8: Risks of dementia in males with individual LTCs at baseline** | 37 |
| **Supplementary Table 9: Risks of dementia in females with individual LTCs at baseline** | 39 |
| **Supplementary Table 10: Risks of dementia in the mid-aged with individual LTCs at baseline** | 41 |
| **Supplementary Table 11: Risks of dementia in the old-aged with individual LTCs at baseline** | 43 |
| **Supplementary Table 12: Risks of dementia in *APOE4* non-carriers with individual LTCs at baseline** | 45 |
| **Supplementary Table 13: Risks of dementia in *APOE4* carriers with individual LTCs at baseline** | 47 |
| **Supplementary Table 14: Subgroup analyses for the association between multimorbidity status and dementia by sex** | 49 |
| **Supplementary Table 15: Subgroup analyses for the association between multimorbidity status and dementia by age** | 50 |
| **Supplementary Table 16: Subgroup analyses for the association between multimorbidity status and dementia by *APOE4* status** | 51 |

**Supplementary Table 1: Description of ICD-10 codes included in each LTC category in the UKB**

| **LTC categories** | **Abbreviations** | **ICD-10 codes** | **Diseases** |
| --- | --- | --- | --- |
| **Allergy** | **-** | J301 | Allergic rhinitis due to pollen |
|  |  | J302 | Other seasonal allergic rhinitis |
|  |  | J303 | Other allergic rhinitis |
|  |  | J304 | Allergic rhinitis, unspecified |
|  |  | J450 | Predominantly allergic asthma |
|  |  | K522 | Allergic and dietetic gastroenteritis and colitis |
|  |  | L20 | Atopic dermatitis |
|  |  | L23 | Allergic contact dermatitis |
|  |  | L500 | Allergic urticaria |
|  |  | Z516 | Desensitization to allergens |
| **Anemia** | **-** | D50 | Iron deficiency anaemia |
|  |  | D51 | Vitamin B12 deficiency anaemia |
|  |  | D52 | Folate deficiency anaemia |
|  |  | D53 | Other nutritional anaemias |
|  |  | D55 | Anaemia due to enzyme disorders |
|  |  | D56 | Thalassaemia |
|  |  | D57 | Sickle-cell disorders |
|  |  | D58 | Other hereditary haemolytic anaemias |
|  |  | D59 | Acquired haemolytic anaemia |
|  |  | D60 | Acquired pure red cell aplasia [erythroblastopenia] |
|  |  | D61 | Other aplastic anaemias |
|  |  | D63 | Anaemia in chronic diseases classified elsewhere |
|  |  | D64 | Other anaemias |
| **Asthma** | **-** | J45 | Asthma |
| **Atrial fibrillation** | **AF** | I48 | Atrial fibrillation and flutter |
| **Autoimmune diseases** | **-** | I731 | Thromboangiitis obliterans [Buerger] |
|  |  | L10 | Pemphigus |
|  |  | L12 | Pemphigoid |
|  |  | L40 | Psoriasis |
|  |  | L41 | Parapsoriasis |
|  |  | L93 | Lupus erythematosus |
|  |  | L94 | Other localized connective tissue disorders |
|  |  | L95 | Vasculitis limited to skin, not elsewhere classified |
|  |  | M30 | Polyarteritis nodosa and related conditions |
|  |  | M31 | Other necrotizing vasculopathies |
|  |  | M32 | Systemic lupus erythematosus |
|  |  | M33 | Dermatopolymyositis |
|  |  | M34 | Systemic sclerosis |
|  |  | M35 | Other systemic involvement of connective tissue |
|  |  | M36 | Systemic disorders of connective tissue in diseases classified elsewhere |
| **Blindness and visual impairment** | **Blindness and VI** | H54 | Visual impairment including blindness (binocular or monocular) |
|  |  | Z442 | Fitting and adjustment of artificial eye |
|  |  | Z970 | Presence of artificial eye |
| **Blood and blood forming organ diseases** | **-** | D66 | Hereditary factor VIII deficiency |
|  |  | D67 | Hereditary factor IX deficiency |
|  |  | D68 | Other coagulation defects |
|  |  | D69 | Purpura and other haemorrhagic conditions |
|  |  | D71 | Functional disorders of polymorphonuclear neutrophils |
|  |  | D720 | Genetic anomalies of leukocytes |
|  |  | D730 | Hyposplenism |
|  |  | D731 | Hypersplenism |
|  |  | D732 | Chronic congestive splenomegaly |
|  |  | D74 | Methaemoglobinaemia |
|  |  | D750 | Familial erythrocytosis |
|  |  | D761 | Haemophagocytic lymphohistiocytosis |
|  |  | D763 | Other histiocytosis syndromes |
|  |  | D77 | Other disorders of blood and blood-forming organs in diseases classified elsewhere |
|  |  | D80 | Immunodeficiency with predominantly antibody defects |
|  |  | D81 | Combined immunodeficiencies |
|  |  | D82 | Immunodeficiency associated with other major defects |
|  |  | D83 | Common variable immunodeficiency |
|  |  | D84 | Other immunodeficiencies |
|  |  | D86 | Sarcoidosis |
|  |  | D89 | Other disorders involving the immune mechanism, not elsewhere classified |
| **Bradycardias and conduction diseases** | **-** | I441 | Atrioventricular block, second degree |
|  |  | I442 | Atrioventricular block, complete |
|  |  | I443 | Other and unspecified atrioventricular block |
|  |  | I453 | Trifascicular block |
|  |  | I455 | Other specified heart block |
|  |  | Z950 | Presence of cardiac pacemaker |
| **Cardiac valve diseases** | **-** | I05 | Rheumatic mitral valve diseases |
|  |  | I06 | Rheumatic aortic valve diseases |
|  |  | I07 | Rheumatic tricuspid valve diseases |
|  |  | I08 | Multiple valve diseases |
|  |  | I091 | Rheumatic diseases of endocardium, valve unspecified |
|  |  | I098 | Other specified rheumatic heart diseases |
|  |  | I34 | Nonrheumatic mitral valve disorders |
|  |  | I35 | Nonrheumatic aortic valve disorders |
|  |  | I36 | Nonrheumatic tricuspid valve disorders |
|  |  | I37 | Pulmonary valve disorders |
|  |  | I38 | Endocarditis, valve unspecified |
|  |  | I390 | Mitral valve disorders in diseases classified elsewhere |
|  |  | I391 | Aortic valve disorders in diseases classified elsewhere |
|  |  | I392 | Tricuspid valve disorders in diseases classified elsewhere |
|  |  | I393 | Pulmonary valve disorders in diseases classified elsewhere |
|  |  | I394 | Multiple valve disorders in diseases classified elsewhere |
|  |  | Q22 | Congenital malformations of pulmonary and tricuspid valves |
|  |  | Q23 | Congenital malformations of aortic and mitral valves |
|  |  | Z952 | Presence of prosthetic heart valve |
|  |  | Z953 | Presence of xenogenic heart valve |
|  |  | Z954 | Presence of other heart-valve replacement |
| **Cataract and other lens diseases** | **-** | H25 | Senile cataract |
|  |  | H26 | Other cataract |
|  |  | H27 | Other disorders of lens |
|  |  | H28 | Cataract and other disorders of lens in diseases classified elsewhere |
|  |  | Q12 | Congenital lens malformations |
|  |  | Z961 | Presence of intraocular lens |
| **Cerebrovascular disease** | **CVD** | G45 | Transient cerebral ischaemic attacks and related syndromes |
|  |  | G46 | Vascular syndromes of brain in cerebrovascular diseases |
|  |  | I60 | Subarachnoid haemorrhage |
|  |  | I61 | Intracerebral haemorrhage |
|  |  | I62 | Other nontraumatic intracranial haemorrhage |
|  |  | I63 | Cerebral infarction |
|  |  | I64 | Stroke, not specified as haemorrhage or infarction |
|  |  | I67 | Other cerebrovascular diseases |
|  |  | I69 | Sequelae of cerebrovascular disease |
| **Chromosomal abnormalities** | **-** | Q90 | Down syndrome |
|  |  | Q91 | Edwards syndrome and Patau syndrome |
|  |  | Q92 | Other trisomies and partial trisomies of the autosomes, not elsewhere classified |
|  |  | Q93 | Monosomies and deletions from the autosomes, not elsewhere classified |
|  |  | Q95 | Balanced rearrangements and structural markers, not elsewhere classified |
|  |  | Q96 | Turner syndrome |
|  |  | Q97 | Other sex chromosome abnormalities, female phenotype, not elsewhere classified |
|  |  | Q98 | Other sex chromosome abnormalities, male phenotype, not elsewhere classified |
|  |  | Q99 | Other chromosome abnormalities, not elsewhere classified |
| **Chronic infectious diseases** | **-** | A15 | Respiratory tuberculosis, bacteriologically and histologically confirmed |
|  |  | A16 | Respiratory tuberculosis, not confirmed bacteriologically or histologically |
|  |  | A17 | Tuberculosis of nervous system |
|  |  | A18 | Tuberculosis of other organs |
|  |  | A19 | Miliary tuberculosis |
|  |  | A30 | Leprosy [Hansen disease] |
|  |  | A31 | Infection due to other mycobacteria |
|  |  | A50 | Congenital syphilis |
|  |  | A52 | Late syphilis |
|  |  | A53 | Other and unspecified syphilis |
|  |  | A65 | Nonvenereal syphilis |
|  |  | A66 | Yaws |
|  |  | A67 | Pinta [carate] |
|  |  | A692 | Lyme disease |
|  |  | A81 | Atypical virus infections of central nervous system |
|  |  | B20 | Human immunodeficiency virus [HIV] disease resulting in infectious and parasitic diseases |
|  |  | B21 | Human immunodeficiency virus [HIV] disease resulting in malignant neoplasms |
|  |  | B22 | Human immunodeficiency virus [HIV] disease resulting in other specified diseases |
|  |  | B23 | Human immunodeficiency virus [HIV] disease resulting in other conditions |
|  |  | B24 | Unspecified human immunodeficiency virus [HIV] disease |
|  |  | B381 | Chronic pulmonary coccidioidomycosis |
|  |  | B391 | Chronic pulmonary histoplasmosis capsulati |
|  |  | B401 | Chronic pulmonary blastomycosis |
|  |  | B572 | Chagas disease (chronic) with heart involvement |
|  |  | B573 | Chagas disease (chronic) with digestive system involvement |
|  |  | B574 | Chagas disease (chronic) with nervous system involvement |
|  |  | B575 | Chagas disease (chronic) with other organ involvement |
|  |  | B65 | Schistosomiasis [bilharziasis] |
|  |  | B92 | Sequelae of leprosy |
|  |  | B94 | Sequelae of other and unspecified infectious and parasitic diseases |
|  |  | J65 | Pneumoconiosis associated with tuberculosis |
|  |  | M863 | Chronic multifocal osteomyelitis |
|  |  | M864 | Chronic osteomyelitis with draining sinus |
|  |  | M865 | Other chronic haematogenous osteomyelitis |
|  |  | M866 | Other chronic osteomyelitis |
| **Chronic kidney disease** | **CKD** | I120 | Hypertensive renal disease with renal failure |
|  |  | I130 | Hypertensive heart and renal disease with (congestive) heart failure |
|  |  | I131 | Hypertensive heart and renal disease with renal failure |
|  |  | I132 | Hypertensive heart and renal disease with both (congestive) heart failure and renal failure |
|  |  | I139 | Hypertensive heart and renal disease, unspecified |
|  |  | N01 | Rapidly progressive nephritic syndrome |
|  |  | N03 | Chronic nephritic syndrome |
|  |  | N04 | Nephrotic syndrome |
|  |  | N05 | Unspecified nephritic syndrome |
|  |  | N07 | Hereditary nephropathy, not elsewhere classified |
|  |  | N08 | Glomerular disorders in diseases classified elsewhere |
|  |  | N11 | Chronic tubulo-interstitial nephritis |
|  |  | N183 | Chronic kidney disease, stage 3 |
|  |  | N184 | Chronic kidney disease, stage 4 |
|  |  | N185 | Chronic kidney disease, stage 5 |
|  |  | N189 | Chronic kidney disease, unspecified |
|  |  | Q60 | Renal agenesis and other reduction defects of kidney |
|  |  | Q611 | Polycystic kidney, autosomal recessive |
|  |  | Q612 | Polycystic kidney, autosomal dominant |
|  |  | Q613 | Polycystic kidney, unspecified |
|  |  | Q614 | Renal dysplasia |
|  |  | Q615 | Medullary cystic kidney |
|  |  | Q618 | Other cystic kidney diseases |
|  |  | Q619 | Cystic kidney disease, unspecified |
|  |  | Z905 | Acquired absence of kidney |
|  |  | Z940 | Kidney transplant status |
| **Chronic liver diseases** | **CLD** | B18 | Chronic viral hepatitis |
|  |  | K70 | Alcoholic liver disease |
|  |  | K713 | Toxic liver disease with chronic persistent hepatitis |
|  |  | K714 | Toxic liver disease with chronic lobular hepatitis |
|  |  | K715 | Toxic liver disease with chronic active hepatitis |
|  |  | K717 | Toxic liver disease with fibrosis and cirrhosis of liver |
|  |  | K721 | Chronic hepatic failure |
|  |  | K73 | Chronic hepatitis, not elsewhere classified |
|  |  | K74 | Fibrosis and cirrhosis of liver |
|  |  | K753 | Granulomatous hepatitis, not elsewhere classified |
|  |  | K754 | Autoimmune hepatitis |
|  |  | K758 | Other specified inflammatory liver diseases |
|  |  | K761 | Chronic passive congestion of liver |
|  |  | K766 | Portal hypertension |
|  |  | K767 | Hepatorenal syndrome |
|  |  | K778 | Liver disorders in other diseases classified elsewhere |
|  |  | Q446 | Cystic disease of liver |
|  |  | Z944 | Liver transplant status |
| **Chronic pancreas, biliary tract and gallbladder diseases** | **-** | K800 | Calculus of gallbladder with acute cholecystitis |
|  |  | K801 | Calculus of gallbladder with other cholecystitis |
|  |  | K802 | Calculus of gallbladder without cholecystitis |
|  |  | K808 | Other cholelithiasis |
|  |  | K811 | Chronic cholecystitis |
|  |  | K86 | Other diseases of pancreas |
|  |  | Q440 | Agenesis, aplasia and hypoplasia of gallbladder |
|  |  | Q441 | Other congenital malformations of gallbladder |
|  |  | Q442 | Atresia of bile ducts |
|  |  | Q443 | Congenital stenosis and stricture of bile ducts |
|  |  | Q444 | Choledochal cyst |
|  |  | Q445 | Other congenital malformations of bile ducts |
|  |  | Q450 | Agenesis, aplasia and hypoplasia of pancreas |
| **Chronic ulcer of the skin** | **-** | I830 | Varicose veins of lower extremities with ulcer |
|  |  | I832 | Varicose veins of lower extremities with both ulcer and inflammation |
|  |  | L89 | Decubitus ulcer and pressure area |
|  |  | L97 | Ulcer of lower limb, not elsewhere classified |
|  |  | L984 | Chronic ulcer of skin, not elsewhere classified |
| **Colitis and related diseases** | **-** | K520 | Gastroenteritis and colitis due to radiation |
|  |  | K528 | Other specified noninfective gastroenteritis and colitis |
|  |  | K551 | Chronic vascular disorders of intestine |
|  |  | K552 | Angiodysplasia of colon |
|  |  | K572 | Diverticular disease of large intestine with perforation and abscess |
|  |  | K573 | Diverticular disease of large intestine without perforation or abscess |
|  |  | K574 | Diverticular disease of both small and large intestine with perforation and abscess |
|  |  | K575 | Diverticular disease of both small and large intestine without perforation or abscess |
|  |  | K578 | Diverticular disease of intestine, part unspecified, with perforation and abscess |
|  |  | K579 | Diverticular disease of intestine, part unspecified, without perforation or abscess |
|  |  | K58 | Irritable bowel syndrome |
|  |  | K590 | Constipation |
|  |  | K592 | Neurogenic bowel, not elsewhere classified |
|  |  | K62 | Other diseases of anus and rectum |
|  |  | K634 | Enteroptosis |
|  |  | K64 | Haemorrhoids and perianal venous thrombosis |
| **Chronic obstructive pulmonary disease, chronic bronchitis and emphysema** | **COPD, CB and emphysema** | J41 | Simple and mucopurulent chronic bronchitis |
|  |  | J42 | Unspecified chronic bronchitis |
|  |  | J43 | Emphysema |
|  |  | J44 | Other chronic obstructive pulmonary disease |
|  |  | J47 | Bronchiectasis |
| **Deafness and hearing impairment** | **Deafness and HI** | H80 | Otosclerosis |
|  |  | H90 | Conductive and sensorineural hearing loss |
|  |  | H911 | Presbycusis |
|  |  | H913 | Deaf mutism, not elsewhere classified |
|  |  | H919 | Hearing loss, unspecified |
|  |  | Q16 | Congenital malformations of ear causing impairment of hearing |
|  |  | Z453 | Adjustment and management of implanted hearing device |
|  |  | Z461 | Fitting and adjustment of hearing aid |
|  |  | Z962 | Presence of otological and audiological implants |
|  |  | Z974 | Presence of external hearing-aid |
| **Degenerative joint diseases (e.g. osteoarthritis)** | **Degenerative joint diseases (e.g. OA)** | M15 | Polyarthrosis |
|  |  | M16 | Coxarthrosis [arthrosis of hip] |
|  |  | M17 | Gonarthrosis [arthrosis of knee] |
|  |  | M18 | Arthrosis of first carpometacarpal joint |
|  |  | M19 | Other arthrosis |
|  |  | M362 | Haemophilic arthropathy |
|  |  | M363 | Arthropathy in other blood disorders |
| **Depression and mood diseases** | **-** | F30 | Manic episode |
|  |  | F31 | Bipolar affective disorder |
|  |  | F32 | Depressive episode |
|  |  | F33 | Recurrent depressive disorder |
|  |  | F34 | Persistent mood [affective] disorders |
|  |  | F38 | Other mood [affective] disorders |
|  |  | F39 | Unspecified mood [affective] disorder |
|  |  | F412 | Mixed anxiety and depressive disorder |
| **Diabetes** | **-** | E10 | Insulin-dependent diabetes mellitus |
|  |  | E11 | Non-insulin-dependent diabetes mellitus |
|  |  | E13 | Other specified diabetes mellitus |
|  |  | E14 | Unspecified diabetes mellitus |
|  |  | E891 | Postprocedural hypoinsulinaemia |
| **Dorsopathies** | **-** | M40 | Kyphosis and lordosis |
|  |  | M41 | Scoliosis |
|  |  | M42 | Spinal osteochondrosis |
|  |  | M43 | Other deforming dorsopathies |
|  |  | M47 | Spondylosis |
|  |  | M48 | Other spondylopathies |
|  |  | M49 | Spondylopathies in diseases classified elsewhere |
|  |  | M50 | Cervical disc disorders |
|  |  | M51 | Other intervertebral disc disorders |
|  |  | M53 | Other dorsopathies, not elsewhere classified |
|  |  | Q675 | Congenital deformity of spine |
|  |  | Q761 | Klippel-Feil syndrome |
|  |  | Q764 | Other congenital malformations of spine, not associated with scoliosis |
| **Dyslipidemia** | **-** | E78 | Disorders of lipoprotein metabolism and other lipidaemias |
| **Ear, nose and throat diseases** | **-** | H604 | Cholesteatoma of external ear |
|  |  | H661 | Chronic tubotympanic suppurative otitis media |
|  |  | H662 | Chronic atticoantral suppurative otitis media |
|  |  | H663 | Other chronic suppurative otitis media |
|  |  | H701 | Chronic mastoiditis |
|  |  | H71 | Cholesteatoma of middle ear |
|  |  | H731 | Chronic myringitis |
|  |  | H741 | Adhesive middle ear disease |
|  |  | H810 | MÚniÞre disease |
|  |  | H831 | Labyrinthine fistula |
|  |  | H832 | Labyrinthine dysfunction |
|  |  | H95 | Postprocedural disorders of ear and mastoid process, not elsewhere classified |
|  |  | J300 | Vasomotor rhinitis |
|  |  | J31 | Chronic rhinitis, nasopharyngitis and pharyngitis |
|  |  | J32 | Chronic sinusitis |
|  |  | J33 | Nasal polyp |
|  |  | J341 | Cyst and mucocele of nose and nasal sinus |
|  |  | J342 | Deviated nasal septum |
|  |  | J343 | Hypertrophy of nasal turbinates |
|  |  | J35 | Chronic diseases of tonsils and adenoids |
|  |  | J37 | Chronic laryngitis and laryngotracheitis |
|  |  | J380 | Paralysis of vocal cords and larynx |
|  |  | J386 | Stenosis of larynx |
|  |  | K051 | Chronic gingivitis |
|  |  | K053 | Chronic periodontitis |
|  |  | K07 | Dentofacial anomalies [including malocclusion] |
|  |  | K110 | Atrophy of salivary gland |
|  |  | K117 | Disturbances of salivary secretion |
|  |  | Q30 | Congenital malformations of nose |
|  |  | Q31 | Congenital malformations of larynx |
|  |  | Q32 | Congenital malformations of trachea and bronchus |
|  |  | Q35 | Cleft palate |
|  |  | Q36 | Cleft lip |
|  |  | Q37 | Cleft palate with cleft lip |
|  |  | Q38 | Other congenital malformations of tongue, mouth and pharynx |
| **Epilepsy** | **-** | G40 | Epilepsy |
| **Esophagus, stomach and duodenum diseases** | **-** | I85 | Oesophageal varices |
|  |  | I864 | Gastric varices |
|  |  | I982 | Oesophageal varices without bleeding in diseases classified elsewhere |
|  |  | I983 | Oesophageal varices with bleeding in diseases classified elsewhere |
|  |  | K21 | Gastro-oesophageal reflux disease |
|  |  | K220 | Achalasia of cardia |
|  |  | K222 | Oesophageal obstruction |
|  |  | K224 | Dyskinesia of oesophagus |
|  |  | K225 | Diverticulum of oesophagus, acquired |
|  |  | K227 | Barrett oesophagus |
|  |  | K230 | Tuberculous oesophagitis |
|  |  | K231 | Megaoesophagus in Chagas disease |
|  |  | K254 | Gastric ulcer: Chronic or unspecified with haemorrhage |
|  |  | K255 | Gastric ulcer: Chronic or unspecified with perforation |
|  |  | K256 | Gastric ulcer: Chronic or unspecified with both haemorrhage and perforation |
|  |  | K257 | Gastric ulcer: Chronic without haemorrhage or perforation |
|  |  | K264 | Duodenal ulcer: Chronic or unspecified with haemorrhage |
|  |  | K265 | Duodenal ulcer: Chronic or unspecified with perforation |
|  |  | K266 | Duodenal ulcer: Chronic or unspecified with both haemorrhage and perforation |
|  |  | K267 | Duodenal ulcer: Chronic without haemorrhage or perforation |
|  |  | K274 | Peptic ulcer, site unspecified: Chronic or unspecified with haemorrhage |
|  |  | K275 | Peptic ulcer, site unspecified: Chronic or unspecified with perforation |
|  |  | K276 | Peptic ulcer, site unspecified: Chronic or unspecified with both haemorrhage and perforation |
|  |  | K277 | Peptic ulcer, site unspecified: Chronic without haemorrhage or perforation |
|  |  | K284 | Gastrojejunal ulcer: Chronic or unspecified with haemorrhage |
|  |  | K285 | Gastrojejunal ulcer: Chronic or unspecified with perforation |
|  |  | K286 | Gastrojejunal ulcer: Chronic or unspecified with both haemorrhage and perforation |
|  |  | K287 | Gastrojejunal ulcer: Chronic without haemorrhage or perforation |
|  |  | K293 | Chronic superficial gastritis |
|  |  | K294 | Chronic atrophic gastritis |
|  |  | K295 | Chronic gastritis, unspecified |
|  |  | K296 | Other gastritis |
|  |  | K297 | Gastritis, unspecified |
|  |  | K298 | Duodenitis |
|  |  | K299 | Gastroduodenitis, unspecified |
|  |  | K311 | Adult hypertrophic pyloric stenosis |
|  |  | K312 | Hourglass stricture and stenosis of stomach |
|  |  | K313 | Pylorospasm, not elsewhere classified |
|  |  | K314 | Gastric diverticulum |
|  |  | K315 | Obstruction of duodenum |
|  |  | Q39 | Congenital malformations of oesophagus |
|  |  | Q40 | Other congenital malformations of upper alimentary tract |
|  |  | Z903 | Acquired absence of part of stomach |
| **Glaucoma** | **-** | H401 | Primary open-angle glaucoma |
|  |  | H402 | Primary angle-closure glaucoma |
|  |  | H403 | Glaucoma secondary to eye trauma |
|  |  | H404 | Glaucoma secondary to eye inflammation |
|  |  | H405 | Glaucoma secondary to other eye disorders |
|  |  | H406 | Glaucoma secondary to drugs |
|  |  | H408 | Other glaucoma |
|  |  | H409 | Glaucoma, unspecified |
| **Heart failure** | **HF** | I110 | Hypertensive heart disease with (congestive) heart failure |
|  |  | I130 | Hypertensive heart and renal disease with (congestive) heart failure |
|  |  | I132 | Hypertensive heart and renal disease with both (congestive) heart failure and renal failure |
|  |  | I27 | Other pulmonary heart diseases |
|  |  | I280 | Arteriovenous fistula of pulmonary vessels |
|  |  | I42 | Cardiomyopathy |
|  |  | I43 | Cardiomyopathy in diseases classified elsewhere |
|  |  | I50 | Heart failure |
|  |  | I515 | Myocardial degeneration |
|  |  | I517 | Cardiomegaly |
|  |  | I528 | Other heart disorders in other diseases classified elsewhere |
|  |  | Z941 | Heart transplant status |
|  |  | Z943 | Heart and lungs transplant status |
| **Hematological neoplasms** | **HN** | C81 | Hodgkin lymphoma |
|  |  | C82 | Follicular lymphoma |
|  |  | C83 | Non-follicular lymphoma |
|  |  | C84 | Mature T/NK-cell lymphomas |
|  |  | C85 | Other and unspecified types of non-Hodgkin lymphoma |
|  |  | C86 | Other specified types of T/NK-cell lymphoma |
|  |  | C88 | Malignant immunoproliferative diseases |
|  |  | C90 | Multiple myeloma and malignant plasma cell neoplasms |
|  |  | C91 | Lymphoid leukaemia |
|  |  | C92 | Myeloid leukaemia |
|  |  | C93 | Monocytic leukaemia |
|  |  | C94 | Other leukaemias of specified cell type |
|  |  | C95 | Leukaemia of unspecified cell type |
|  |  | C96 | Other and unspecified malignant neoplasms of lymphoid, haematopoietic and related tissue |
| **Hypertension** | **-** | I10 | Essential (primary) hypertension |
|  |  | I11 | Hypertensive heart disease |
|  |  | I12 | Hypertensive renal disease |
|  |  | I13 | Hypertensive heart and renal disease |
|  |  | I15 | Secondary hypertension |
| **Inflammatory arthropathies** | **-** | M023 | Reiter disease |
|  |  | M05 | Seropositive rheumatoid arthritis |
|  |  | M06 | Other rheumatoid arthritis |
|  |  | M07 | Psoriatic and enteropathic arthropathies |
|  |  | M08 | Juvenile arthritis |
|  |  | M09 | Juvenile arthritis in diseases classified elsewhere |
|  |  | M10 | Gout |
|  |  | M11 | Other crystal arthropathies |
|  |  | M12 | Other specific arthropathies |
|  |  | M13 | Other arthritis |
|  |  | M14 | Arthropathies in other diseases classified elsewhere |
|  |  | M45 | Ankylosing spondylitis |
|  |  | M460 | Spinal enthesopathy |
|  |  | M461 | Sacroiliitis, not elsewhere classified |
|  |  | M468 | Other specified inflammatory spondylopathies |
|  |  | M469 | Inflammatory spondylopathy, unspecified |
| **Inflammatory bowel disease** | **IBD** | K50 | Crohn disease [regional enteritis] |
|  |  | K51 | Ulcerative colitis |
| **Ischemic heart disease** | **IHD** | I20 | Angina pectoris |
|  |  | I21 | Acute myocardial infarction |
|  |  | I22 | Subsequent myocardial infarction |
|  |  | I24 | Other acute ischaemic heart diseases |
|  |  | I25 | Chronic ischaemic heart disease |
|  |  | Z951 | Presence of aortocoronary bypass graft |
|  |  | Z955 | Presence of coronary angioplasty implant and graft |
| **Migraine and facial pain syndromes** | **-** | G43 | Migraine |
|  |  | G440 | Cluster headache syndrome |
|  |  | G441 | Vascular headache, not elsewhere classified |
|  |  | G442 | Tension-type headache |
|  |  | G443 | Chronic post-traumatic headache |
|  |  | G448 | Other specified headache syndromes |
|  |  | G50 | Disorders of trigeminal nerve |
| **Multiple sclerosis** | **MS** | G35 | Multiple sclerosis |
| **Neurotic, stress-related and somatoform diseases** | **-** | F40 | Phobic anxiety disorders |
|  |  | F41 | Other anxiety disorders |
|  |  | F42 | Obsessive-compulsive disorder |
|  |  | F43 | Reaction to severe stress, and adjustment disorders |
|  |  | F44 | Dissociative [conversion] disorders |
|  |  | F45 | Somatoform disorders |
|  |  | F48 | Other neurotic disorders |
| **Obesity** | **-** | E66 | Obesity ^a^ |
| **Osteoporosis** | **-** | M80 | Osteoporosis with pathological fracture |
|  |  | M81 | Osteoporosis without pathological fracture |
|  |  | M82 | Osteoporosis in diseases classified elsewhere |
| **Other cardiovascular diseases** | **-** | I09 | Other rheumatic heart diseases |
|  |  | I281 | Aneurysm of pulmonary artery |
|  |  | I310 | Chronic adhesive pericarditis |
|  |  | I311 | Chronic constrictive pericarditis |
|  |  | I456 | Pre-excitation syndrome |
|  |  | I495 | Sick sinus syndrome |
|  |  | I498 | Other specified cardiac arrhythmias |
|  |  | I70 | Atherosclerosis |
|  |  | I71 | Aortic aneurysm and dissection |
|  |  | I72 | Other aneurysm and dissection |
|  |  | I790 | Aneurysm of aorta in diseases classified elsewhere |
|  |  | I791 | Aortitis in diseases classified elsewhere |
|  |  | I950 | Idiopathic hypotension |
|  |  | I951 | Orthostatic hypotension |
|  |  | I958 | Other hypotension |
|  |  | Q20 | Congenital malformations of cardiac chambers and connections |
|  |  | Q21 | Congenital malformations of cardiac septa |
|  |  | Q24 | Other congenital malformations of heart |
|  |  | Q25 | Congenital malformations of great arteries |
|  |  | Q26 | Congenital malformations of great veins |
|  |  | Q27 | Other congenital malformations of peripheral vascular system |
|  |  | Q28 | Other congenital malformations of circulatory system |
|  |  | Z958 | Presence of other cardiac and vascular implants and grafts |
|  |  | Z959 | Presence of cardiac and vascular implant and graft, unspecified |
| **Other digestive diseases** | **-** | K660 | Peritoneal adhesions |
|  |  | K900 | Coeliac disease |
|  |  | K901 | Tropical sprue |
|  |  | K902 | Blind loop syndrome, not elsewhere classified |
|  |  | K911 | Postgastric surgery syndromes |
|  |  | K93 | Disorders of other digestive organs in diseases classified elsewhere |
|  |  | Q41 | Congenital absence, atresia and stenosis of small intestine |
|  |  | Q42 | Congenital absence, atresia and stenosis of large intestine |
|  |  | Q43 | Other congenital malformations of intestine |
|  |  | R15 | Faecal incontinence |
|  |  | Z904 | Acquired absence of other parts of digestive tract |
|  |  | Z980 | Intestinal bypass and anastomosis status |
| **Other eye diseases** | **-** | H022 | Lagophthalmos |
|  |  | H023 | Blepharochalasis |
|  |  | H024 | Ptosis of eyelid |
|  |  | H025 | Other disorders affecting eyelid function |
|  |  | H04 | Disorders of lacrimal system |
|  |  | H05 | Disorders of orbit |
|  |  | H104 | Chronic conjunctivitis |
|  |  | H17 | Corneal scars and opacities |
|  |  | H184 | Corneal degeneration |
|  |  | H185 | Hereditary corneal dystrophies |
|  |  | H186 | Keratoconus |
|  |  | H187 | Other corneal deformities |
|  |  | H188 | Other specified disorders of cornea |
|  |  | H189 | Disorder of cornea, unspecified |
|  |  | H193 | Keratitis and keratoconjunctivitis in other diseases classified elsewhere |
|  |  | H198 | Other disorders of sclera and cornea in diseases classified elsewhere |
|  |  | H201 | Chronic iridocyclitis |
|  |  | H21 | Other disorders of iris and ciliary body |
|  |  | H310 | Chorioretinal scars |
|  |  | H311 | Choroidal degeneration |
|  |  | H312 | Hereditary choroidal dystrophy |
|  |  | H318 | Other specified disorders of choroid |
|  |  | H319 | Disorder of choroid, unspecified |
|  |  | H33 | Retinal detachments and breaks |
|  |  | H352 | Other proliferative retinopathy |
|  |  | H353 | Degeneration of macula and posterior pole |
|  |  | H354 | Peripheral retinal degeneration |
|  |  | H355 | Hereditary retinal dystrophy |
|  |  | H357 | Separation of retinal layers |
|  |  | H358 | Other specified retinal disorders |
|  |  | H359 | Retinal disorder, unspecified |
|  |  | H36 | Retinal disorders in diseases classified elsewhere |
|  |  | H47 | Other disorders of optic [2nd] nerve and visual pathways |
|  |  | H48 | Disorders of optic [2nd] nerve and visual pathways in diseases classified elsewhere |
|  |  | H49 | Paralytic strabismus |
|  |  | H51 | Other disorders of binocular movement |
|  |  | Q10 | Congenital malformations of eyelid, lacrimal apparatus and orbit |
|  |  | Q11 | Anophthalmos, microphthalmos and macrophthalmos |
|  |  | Q13 | Congenital malformations of anterior segment of eye |
|  |  | Q14 | Congenital malformations of posterior segment of eye |
|  |  | Q15 | Other congenital malformations of eye |
|  |  | Z947 | Corneal transplant status |
| **Other genitourinary diseases** | **-** | B901 | Sequelae of genitourinary tuberculosis |
|  |  | N200 | Calculus of kidney |
|  |  | N202 | Calculus of kidney with calculus of ureter |
|  |  | N209 | Urinary calculus, unspecified |
|  |  | N210 | Calculus in bladder |
|  |  | N218 | Other lower urinary tract calculus |
|  |  | N219 | Calculus of lower urinary tract, unspecified |
|  |  | N22 | Calculus of urinary tract in diseases classified elsewhere |
|  |  | N301 | Interstitial cystitis (chronic) |
|  |  | N302 | Other chronic cystitis |
|  |  | N303 | Trigonitis |
|  |  | N304 | Irradiation cystitis |
|  |  | N31 | Neuromuscular dysfunction of bladder, not elsewhere classified |
|  |  | N320 | Bladder-neck obstruction |
|  |  | N323 | Diverticulum of bladder |
|  |  | N328 | Other specified disorders of bladder |
|  |  | N329 | Bladder disorder, unspecified |
|  |  | N33 | Bladder disorders in diseases classified elsewhere |
|  |  | N35 | Urethral stricture |
|  |  | N393 | Stress incontinence |
|  |  | N394 | Other specified urinary incontinence |
|  |  | N480 | Leukoplakia of penis |
|  |  | N484 | Impotence of organic origin |
|  |  | N489 | Disorder of penis, unspecified |
|  |  | N701 | Chronic salpingitis and oophoritis |
|  |  | N711 | Chronic inflammatory disease of uterus |
|  |  | N731 | Chronic parametritis and pelvic cellulitis |
|  |  | N734 | Female chronic pelvic peritonitis |
|  |  | N736 | Female pelvic peritoneal adhesions |
|  |  | N761 | Subacute and chronic vaginitis |
|  |  | N763 | Subacute and chronic vulvitis |
|  |  | N81 | Female genital prolapse |
|  |  | N88 | Other noninflammatory disorders of cervix uteri |
|  |  | N895 | Stricture and atresia of vagina |
|  |  | N905 | Atrophy of vulva |
|  |  | N952 | Postmenopausal atrophic vaginitis |
|  |  | Q54 | Hypospadias |
|  |  | Q620 | Congenital hydronephrosis |
|  |  | Q621 | Atresia and stenosis of ureter |
|  |  | Q622 | Congenital megaloureter |
|  |  | Q623 | Other obstructive defects of renal pelvis and ureter |
|  |  | Q624 | Agenesis of ureter |
|  |  | Q627 | Congenital vesico-uretero-renal reflux |
|  |  | Q628 | Other congenital malformations of ureter |
|  |  | Q638 | Other specified congenital malformations of kidney |
|  |  | Q639 | Congenital malformation of kidney, unspecified |
|  |  | Q640 | Epispadias |
|  |  | Q641 | Exstrophy of urinary bladder |
|  |  | Q643 | Other atresia and stenosis of urethra and bladder neck |
|  |  | Q644 | Malformation of urachus |
|  |  | Q645 | Congenital absence of bladder and urethra |
|  |  | Q646 | Congenital diverticulum of bladder |
|  |  | Q647 | Other congenital malformations of bladder and urethra |
|  |  | Q648 | Other specified congenital malformations of urinary system |
|  |  | Q649 | Congenital malformation of urinary system, unspecified |
|  |  | Z906 | Acquired absence of other organs of urinary tract |
|  |  | Z907 | Acquired absence of genital organ(s) |
|  |  | Z960 | Presence of urogenital implants |
| **Other metabolic diseases** | **-** | E20 | Hypoparathyroidism |
|  |  | E21 | Hyperparathyroidism and other disorders of parathyroid gland |
|  |  | E22 | Hyperfunction of pituitary gland |
|  |  | E23 | Hypofunction and other disorders of pituitary gland |
|  |  | E24 | Cushing syndrome |
|  |  | E25 | Adrenogenital disorders |
|  |  | E26 | Hyperaldosteronism |
|  |  | E27 | Other disorders of adrenal gland |
|  |  | E28 | Ovarian dysfunction |
|  |  | E29 | Testicular dysfunction |
|  |  | E31 | Polyglandular dysfunction |
|  |  | E34 | Other endocrine disorders |
|  |  | E35 | Disorders of endocrine glands in diseases classified elsewhere |
|  |  | E40 | Kwashiorkor |
|  |  | E41 | Nutritional marasmus |
|  |  | E42 | Marasmic kwashiorkor |
|  |  | E43 | Unspecified severe protein-energy malnutrition |
|  |  | E44 | Protein-energy malnutrition of moderate and mild degree |
|  |  | E45 | Retarded development following protein-energy malnutrition |
|  |  | E46 | Unspecified protein-energy malnutrition |
|  |  | E64 | Sequelae of malnutrition and other nutritional deficiencies |
|  |  | E70 | Disorders of aromatic amino-acid metabolism |
|  |  | E71 | Disorders of branched-chain amino-acid metabolism and fatty-acid metabolism |
|  |  | E72 | Other disorders of amino-acid metabolism |
|  |  | E74 | Other disorders of carbohydrate metabolism |
|  |  | E75 | Disorders of sphingolipid metabolism and other lipid storage disorders |
|  |  | E76 | Disorders of glycosaminoglycan metabolism |
|  |  | E77 | Disorders of glycoprotein metabolism |
|  |  | E79 | Disorders of purine and pyrimidine metabolism |
|  |  | E80 | Disorders of porphyrin and bilirubin metabolism |
|  |  | E83 | Disorders of mineral metabolism |
|  |  | E84 | Cystic fibrosis |
|  |  | E85 | Amyloidosis |
|  |  | E88 | Other metabolic disorders |
|  |  | E89 | Postprocedural endocrine and metabolic disorders, not elsewhere classified |
|  |  | K903 | Pancreatic steatorrhoea |
|  |  | K904 | Malabsorption due to intolerance, not elsewhere classified |
|  |  | K908 | Other intestinal malabsorption |
|  |  | K909 | Intestinal malabsorption, unspecified |
|  |  | K912 | Postsurgical malabsorption, not elsewhere classified |
|  |  | M83 | Adult osteomalacia |
|  |  | M88 | Paget disease of bone [osteitis deformans] |
|  |  | N25 | Disorders resulting from impaired renal tubular function |
| **Other musculoskeletal and joint diseases** | **-** | B902 | Sequelae of tuberculosis of bones and joints |
|  |  | M212 | Flexion deformity |
|  |  | M213 | Wrist or foot drop (acquired) |
|  |  | M214 | Flat foot [pes planus] (acquired) |
|  |  | M215 | Acquired clawhand, clubhand, clawfoot and clubfoot |
|  |  | M216 | Other acquired deformities of ankle and foot |
|  |  | M217 | Unequal limb length (acquired) |
|  |  | M218 | Other specified acquired deformities of limbs |
|  |  | M219 | Acquired deformity of limb, unspecified |
|  |  | M22 | Disorders of patella |
|  |  | M23 | Internal derangement of knee |
|  |  | M24 | Other specific joint derangements |
|  |  | M252 | Flail joint |
|  |  | M253 | Other instability of joint |
|  |  | M357 | Hypermobility syndrome |
|  |  | M61 | Calcification and ossification of muscle |
|  |  | M652 | Calcific tendinitis |
|  |  | M653 | Trigger finger |
|  |  | M654 | Radial styloid tenosynovitis [de Quervain] |
|  |  | M700 | Chronic crepitant synovitis of hand and wrist |
|  |  | M720 | Palmar fascial fibromatosis [Dupuytren] |
|  |  | M722 | Plantar fascial fibromatosis |
|  |  | M724 | Pseudosarcomatous fibromatosis |
|  |  | M750 | Adhesive capsulitis of shoulder |
|  |  | M751 | Rotator cuff syndrome |
|  |  | M753 | Calcific tendinitis of shoulder |
|  |  | M754 | Impingement syndrome of shoulder |
|  |  | M797 | Fibromyalgia |
|  |  | M841 | Nonunion of fracture [pseudarthrosis] |
|  |  | M89 | Other disorders of bone |
|  |  | M91 | Juvenile osteochondrosis of hip and pelvis |
|  |  | M93 | Other osteochondropathies |
|  |  | M94 | Other disorders of cartilage |
|  |  | M96 | Postprocedural musculoskeletal disorders, not elsewhere classified |
|  |  | M99 | Biomechanical lesions, not elsewhere classified |
|  |  | Q65 | Congenital deformities of hip |
|  |  | Q66 | Congenital deformities of feet |
|  |  | Q68 | Other congenital musculoskeletal deformities |
|  |  | Q71 | Reduction defects of upper limb |
|  |  | Q72 | Reduction defects of lower limb |
|  |  | Q73 | Reduction defects of unspecified limb |
|  |  | Q74 | Other congenital malformations of limb(s) |
|  |  | Q77 | Osteochondrodysplasia with defects of growth of tubular bones and spine |
|  |  | Q78 | Other osteochondrodysplasias |
|  |  | Q796 | Ehlers-Danlos syndrome |
|  |  | Q798 | Other congenital malformations of musculoskeletal system |
|  |  | Q87 | Other specified congenital malformation syndromes affecting multiple systems |
|  |  | S382 | Traumatic amputation of external genital organs |
|  |  | S48 | Traumatic amputation of shoulder and upper arm |
|  |  | S58 | Traumatic amputation of forearm |
|  |  | S68 | Traumatic amputation of wrist and hand |
|  |  | S78 | Traumatic amputation of hip and thigh |
|  |  | S88 | Traumatic amputation of lower leg |
|  |  | S98 | Traumatic amputation of ankle and foot |
|  |  | T05 | Traumatic amputations involving multiple body regions |
|  |  | T096 | Traumatic amputation of trunk, level unspecified |
|  |  | T116 | Traumatic amputation of upper limb, level unspecified |
|  |  | T136 | Traumatic amputation of lower limb, level unspecified |
|  |  | T147 | Crushing injury and traumatic amputation of unspecified body region |
|  |  | T90 | Sequelae of injuries of head |
|  |  | T91 | Sequelae of injuries of neck and trunk |
|  |  | T92 | Sequelae of injuries of upper limb |
|  |  | T93 | Sequelae of injuries of lower limb |
|  |  | T94 | Sequelae of injuries involving multiple and unspecified body regions |
|  |  | T95 | Sequelae of burns, corrosions and frostbite |
|  |  | T96 | Sequelae of poisoning by drugs, medicaments and biological substances |
|  |  | T97 | Sequelae of toxic effects of substances chiefly nonmedicinal as to source |
|  |  | T98 | Sequelae of other and unspecified effects of external causes |
|  |  | Z440 | Fitting and adjustment of artificial arm (complete)(partial) |
|  |  | Z441 | Fitting and adjustment of artificial leg (complete)(partial) |
|  |  | Z891 | Acquired absence of hand and wrist |
|  |  | Z892 | Acquired absence of upper limb above wrist |
|  |  | Z893 | Acquired absence of both upper limbs [any level] |
|  |  | Z894 | Acquired absence of foot and ankle |
|  |  | Z895 | Acquired absence of leg at or below knee |
|  |  | Z896 | Acquired absence of leg above knee |
|  |  | Z897 | Acquired absence of both lower limbs [any level, except toes alone] |
|  |  | Z898 | Acquired absence of upper and lower limbs [any level] |
|  |  | Z899 | Acquired absence of limb, unspecified |
|  |  | Z946 | Bone transplant status |
|  |  | Z966 | Presence of orthopaedic joint implants |
|  |  | Z971 | Presence of artificial limb (complete)(partial) |
| **Other neurological diseases** | **-** | B900 | Sequelae of central nervous system tuberculosis |
|  |  | D482 | Neoplasm of uncertain or unknown behaviour: Peripheral nerves and autonomic nervous system |
|  |  | G041 | Tropical spastic paraplegia |
|  |  | G09 | Sequelae of inflammatory diseases of central nervous system |
|  |  | G10 | Huntington disease |
|  |  | G11 | Hereditary ataxia |
|  |  | G12 | Spinal muscular atrophy and related syndromes |
|  |  | G13 | Systemic atrophies primarily affecting central nervous system in diseases classified elsewhere |
|  |  | G24 | Dystonia |
|  |  | G25 | Other extrapyramidal and movement disorders |
|  |  | G26 | Extrapyramidal and movement disorders in diseases classified elsewhere |
|  |  | G32 | Other degenerative disorders of nervous system in diseases classified elsewhere |
|  |  | G37 | Other demyelinating diseases of central nervous system |
|  |  | G51 | Facial nerve disorders |
|  |  | G52 | Disorders of other cranial nerves |
|  |  | G53 | Cranial nerve disorders in diseases classified elsewhere |
|  |  | G70 | Myasthenia gravis and other myoneural disorders |
|  |  | G71 | Primary disorders of muscles |
|  |  | G723 | Periodic paralysis |
|  |  | G724 | Inflammatory myopathy, not elsewhere classified |
|  |  | G728 | Other specified myopathies |
|  |  | G729 | Myopathy, unspecified |
|  |  | G73 | Disorders of myoneural junction and muscle in diseases classified elsewhere |
|  |  | G80 | Cerebral palsy |
|  |  | G81 | Hemiplegia |
|  |  | G82 | Paraplegia and tetraplegia |
|  |  | G83 | Other paralytic syndromes |
|  |  | G90 | Disorders of autonomic nervous system |
|  |  | G91 | Hydrocephalus |
|  |  | G938 | Other specified disorders of brain |
|  |  | G939 | Disorder of brain, unspecified |
|  |  | G95 | Other diseases of spinal cord |
|  |  | G99 | Other disorders of nervous system in diseases classified elsewhere |
|  |  | M471 | Other spondylosis with myelopathy |
|  |  | Q00 | Anencephaly and similar malformations |
|  |  | Q01 | Encephalocele |
|  |  | Q02 | Microcephaly |
|  |  | Q03 | Congenital hydrocephalus |
|  |  | Q04 | Other congenital malformations of brain |
|  |  | Q05 | Spina bifida |
|  |  | Q06 | Other congenital malformations of spinal cord |
|  |  | Q07 | Other congenital malformations of nervous system |
|  |  | Q760 | Spina bifida occulta |
| **Other psychiatric and behavioral diseases** | **-** | F04 | Organic amnesic syndrome, not induced by alcohol and other psychoactive substances |
|  |  | F06 | Other mental disorders due to brain damage and dysfunction and to physical disease |
|  |  | F07 | Personality and behavioural disorders due to brain disease, damage and dysfunction |
|  |  | F09 | Unspecified organic or symptomatic mental disorder |
|  |  | F102 | Mental and behavioural disorders due to use of alcohol: Dependence syndrome |
|  |  | F106 | Mental and behavioural disorders due to use of alcohol: Amnesic syndrome |
|  |  | F107 | Mental and behavioural disorders due to use of alcohol: Residual and late-onset psychotic disorder |
|  |  | F112 | Mental and behavioural disorders due to use of opioids: Dependence syndrome |
|  |  | F116 | Mental and behavioural disorders due to use of opioids: Amnesic syndrome |
|  |  | F117 | Mental and behavioural disorders due to use of opioids: Residual and late-onset psychotic disorder |
|  |  | F122 | Mental and behavioural disorders due to use of cannabinoids: Dependence syndrome |
|  |  | F126 | Mental and behavioural disorders due to use of cannabinoids: Amnesic syndrome |
|  |  | F127 | Mental and behavioural disorders due to use of cannabinoids: Residual and late-onset psychotic disorder |
|  |  | F132 | Mental and behavioural disorders due to use of sedatives or hypnotics: Dependence syndrome |
|  |  | F136 | Mental and behavioural disorders due to use of sedatives or hypnotics: Amnesic syndrome |
|  |  | F137 | Mental and behavioural disorders due to use of sedatives or hypnotics: Residual and late-onset psychotic disorder |
|  |  | F142 | Mental and behavioural disorders due to use of cocaine: Dependence syndrome |
|  |  | F146 | Mental and behavioural disorders due to use of cocaine: Amnesic syndrome |
|  |  | F147 | Mental and behavioural disorders due to use of cocaine: Residual and late-onset psychotic disorder |
|  |  | F152 | Mental and behavioural disorders due to use of other stimulants, including caffeine: Dependence syndrome |
|  |  | F156 | Mental and behavioural disorders due to use of other stimulants, including caffeine: Amnesic syndrome |
|  |  | F157 | Mental and behavioural disorders due to use of other stimulants, including caffeine: Residual and late-onset psychotic disorder |
|  |  | F162 | Mental and behavioural disorders due to use of hallucinogens: Dependence syndrome |
|  |  | F166 | Mental and behavioural disorders due to use of hallucinogens: Amnesic syndrome |
|  |  | F167 | Mental and behavioural disorders due to use of hallucinogens: Residual and late-onset psychotic disorder |
|  |  | F172 | Mental and behavioural disorders due to use of tobacco: Dependence syndrome |
|  |  | F176 | Mental and behavioural disorders due to use of tobacco: Amnesic syndrome |
|  |  | F177 | Mental and behavioural disorders due to use of tobacco: Residual and late-onset psychotic disorder |
|  |  | F182 | Mental and behavioural disorders due to use of volatile solvents: Dependence syndrome |
|  |  | F186 | Mental and behavioural disorders due to use of volatile solvents: Amnesic syndrome |
|  |  | F187 | Mental and behavioural disorders due to use of volatile solvents: Residual and late-onset psychotic disorder |
|  |  | F192 | Mental and behavioural disorders due to multiple drug use and use of other psychoactive substances: Dependence syndrome |
|  |  | F196 | Mental and behavioural disorders due to multiple drug use and use of other psychoactive substances: Amnesic syndrome |
|  |  | F197 | Mental and behavioural disorders due to multiple drug use and use of other psychoactive substances: Residual and late-onset psychotic disorder |
|  |  | F50 | Eating disorders |
|  |  | F52 | Sexual dysfunction, not caused by organic disorder or disease |
|  |  | F60 | Specific personality disorders |
|  |  | F61 | Mixed and other personality disorders |
|  |  | F62 | Enduring personality changes, not attributable to brain damage and disease |
|  |  | F63 | Habit and impulse disorders |
|  |  | F68 | Other disorders of adult personality and behaviour |
|  |  | F70 | Mild mental retardation |
|  |  | F71 | Moderate mental retardation |
|  |  | F72 | Severe mental retardation |
|  |  | F73 | Profound mental retardation |
|  |  | F78 | Other mental retardation |
|  |  | F79 | Unspecified mental retardation |
|  |  | F80 | Specific developmental disorders of speech and language |
|  |  | F81 | Specific developmental disorders of scholastic skills |
|  |  | F82 | Specific developmental disorder of motor function |
|  |  | F83 | Mixed specific developmental disorders |
|  |  | F84 | Pervasive developmental disorders |
|  |  | F88 | Other disorders of psychological development |
|  |  | F89 | Unspecified disorder of psychological development |
|  |  | F95 | Tic disorders |
|  |  | F99 | Mental disorder, not otherwise specified |
| **Other respiratory diseases** | **-** | B909 | Sequelae of respiratory and unspecified tuberculosis |
|  |  | E662 | Extreme obesity with alveolar hypoventilation |
|  |  | J60 | Coalworker pneumoconiosis |
|  |  | J61 | Pneumoconiosis due to asbestos and other mineral fibres |
|  |  | J62 | Pneumoconiosis due to dust containing silica |
|  |  | J63 | Pneumoconiosis due to other inorganic dusts |
|  |  | J64 | Unspecified pneumoconiosis |
|  |  | J65 | Pneumoconiosis associated with tuberculosis |
|  |  | J66 | Airway disease due to specific organic dust |
|  |  | J67 | Hypersensitivity pneumonitis due to organic dust |
|  |  | J684 | Chronic respiratory conditions due to chemicals, gases, fumes and vapours |
|  |  | J701 | Chronic and other pulmonary manifestations due to radiation |
|  |  | J703 | Chronic drug-induced interstitial lung disorders |
|  |  | J704 | Drug-induced interstitial lung disorders, unspecified |
|  |  | J84 | Other interstitial pulmonary diseases |
|  |  | J92 | Pleural plaque |
|  |  | J941 | Fibrothorax |
|  |  | J953 | Chronic pulmonary insufficiency following surgery |
|  |  | J955 | Postprocedural subglottic stenosis |
|  |  | J961 | Chronic respiratory failure |
|  |  | J98 | Other respiratory disorders |
|  |  | Q33 | Congenital malformations of lung |
|  |  | Q34 | Other congenital malformations of respiratory system |
|  |  | Z902 | Acquired absence of lung [part of] |
|  |  | Z942 | Lung transplant status |
|  |  | Z943 | Heart and lungs transplant status |
|  |  | Z963 | Presence of artificial larynx |
| **Other skin diseases** | **-** | L13 | Other bullous disorders |
|  |  | L28 | Lichen simplex chronicus and prurigo |
|  |  | L301 | Dyshidrosis [pompholyx] |
|  |  | L43 | Lichen planus |
|  |  | L508 | Other urticaria |
|  |  | L581 | Chronic radiodermatitis |
|  |  | L85 | Other epidermal thickening |
|  |  | Q80 | Congenital ichthyosis |
|  |  | Q81 | Epidermolysis bullosa |
|  |  | Q821 | Xeroderma pigmentosum |
|  |  | Q822 | Mastocytosis |
|  |  | Q829 | Congenital malformation of skin, unspecified |
| **Parkinson and parkinsonism** | **-** | G20 | Parkinson disease |
|  |  | G21 | Secondary parkinsonism |
|  |  | G22 | Parkinsonism in diseases classified elsewhere |
|  |  | G23 | Other degenerative diseases of basal ganglia |
| **Peripheral neuropathy** | **PN** | B91 | Sequelae of poliomyelitis |
|  |  | G14 | Postpolio syndrome |
|  |  | G54 | Nerve root and plexus disorders |
|  |  | G55 | Nerve root and plexus compressions in diseases classified elsewhere |
|  |  | G56 | Mononeuropathies of upper limb |
|  |  | G57 | Mononeuropathies of lower limb |
|  |  | G58 | Other mononeuropathies |
|  |  | G59 | Mononeuropathy in diseases classified elsewhere |
|  |  | G60 | Hereditary and idiopathic neuropathy |
|  |  | G628 | Other specified polyneuropathies |
|  |  | G629 | Polyneuropathy, unspecified |
|  |  | G63 | Polyneuropathy in diseases classified elsewhere |
|  |  | M472 | Other spondylosis with radiculopathy |
|  |  | M531 | Cervicobrachial syndrome |
|  |  | M541 | Radiculopathy |
| **Peripheral vascular disease** | **PVD** | I702 | Atherosclerosis of arteries of extremities |
|  |  | I73 | Other peripheral vascular diseases |
|  |  | I792 | Peripheral angiopathy in diseases classified elsewhere |
|  |  | I798 | Other disorders of arteries, arterioles and capillaries in diseases classified elsewhere |
| **Prostate diseases** | **-** | N40 | Hyperplasia of prostate |
|  |  | N411 | Chronic prostatitis |
|  |  | N418 | Other inflammatory diseases of prostate |
| **Schizophrenia and delusional diseases** | **-** | F20 | Schizophrenia |
|  |  | F22 | Persistent delusional disorders |
|  |  | F24 | Induced delusional disorder |
|  |  | F25 | Schizoaffective disorders |
|  |  | F28 | Other nonorganic psychotic disorders |
| **Sleep disorders** | **-** | F510 | Nonorganic insomnia |
|  |  | F511 | Nonorganic hypersomnia |
|  |  | F512 | Nonorganic disorder of the sleep-wake schedule |
|  |  | F513 | Sleepwalking [somnambulism] |
|  |  | G47 | Sleep disorders |
| **Solid neoplasms** | **-** | C | Malignant neoplasms |
|  |  | D00 | Carcinoma in situ of oral cavity, oesophagus and stomach |
|  |  | D01 | Carcinoma in situ of other and unspecified digestive organs |
|  |  | D02 | Carcinoma in situ of middle ear and respiratory system |
|  |  | D03 | Melanoma in situ |
|  |  | D04 | Carcinoma in situ of skin |
|  |  | D05 | Carcinoma in situ of breast |
|  |  | D06 | Carcinoma in situ of cervix uteri |
|  |  | D07 | Carcinoma in situ of other and unspecified genital organs |
|  |  | D09 | Carcinoma in situ of other and unspecified sites |
|  |  | D320 | Benign neoplasm: Cerebral meninges |
|  |  | D321 | Benign neoplasm: Spinal meninges |
|  |  | D329 | Benign neoplasm: Meninges, unspecified |
|  |  | D330 | Benign neoplasm: Brain, supratentorial |
|  |  | D331 | Benign neoplasm: Brain, infratentorial |
|  |  | D332 | Benign neoplasm: Brain, unspecified |
|  |  | D333 | Benign neoplasm: Cranial nerves |
|  |  | D334 | Benign neoplasm: Spinal cord |
|  |  | Q85 | Phakomatoses, not elsewhere classified |
| **Thyroid diseases** | **-** | E00 | Congenital iodine-deficiency syndrome |
|  |  | E01 | Iodine-deficiency-related thyroid disorders and allied conditions |
|  |  | E02 | Subclinical iodine-deficiency hypothyroidism |
|  |  | E03 | Other hypothyroidism |
|  |  | E05 | Thyrotoxicosis [hyperthyroidism] |
|  |  | E062 | Chronic thyroiditis with transient thyrotoxicosis |
|  |  | E063 | Autoimmune thyroiditis |
|  |  | E065 | Other chronic thyroiditis |
|  |  | E07 | Other disorders of thyroid |
|  |  | E350 | Disorders of thyroid gland in diseases classified elsewhere |
|  |  | E890 | Postprocedural hypothyroidism |
| **Venous/lymphatic diseases** | **-** | I780 | Hereditary haemorrhagic telangiectasia |
|  |  | I83 | Varicose veins of lower extremities |
|  |  | I87 | Other disorders of veins |
|  |  | I89 | Other noninfective disorders of lymphatic vessels and lymph nodes |
|  |  | I972 | Postmastectomy lymphoedema syndrome |
|  |  | Q820 | Hereditary lymphoedema |

Notes: ^a^ Obesity was defined as a BMI of ≥30 Kg/m^2^.

When all of the sub-codes within a given ICD-10 code were classified as chronic, the highest possible level of aggregation of the hierarchy was included in this list (e.g. three-digit code for asthma [J45], one-digit code for malignant neoplasms [C], etc.) ^1^.

Abbreviations: ICD-10, International Classification of Diseases, Tenth Revision; LTC, long-term condition; UKB, UK Biobank; AF, atrial fibrillation; CB, chronic bronchitis; CKD, chronic kidney disease; CLD, chronic liver diseases; COPD, chronic obstructive pulmonary disease; CVD, cerebrovascular disease; E.g., for example; OA, osteoarthritis; HF, heart failure; HI, hearing impairment; HN, hematological neoplasm; IBD, inflammatory bowel disease; IHD, ischemic heart disease;MS, multiple sclerosis; PN, peripheral neuropathy; PVD, peripheral vascular disease; VI, visual impairment; BMI, body mass index.

**References of Supplementary Table 1**

1. Calderón-Larrañaga A, Vetrano DL, Onder G, et al. Assessing and Measuring Chronic Multimorbidity in the Older Population: A Proposal for Its Operationalization. *The journals of gerontology Series A, Biological sciences and medical sciences* 2017; **72**(10): 1417-23.

**Supplementary Table 2: The** **prevalences of** **LTCs at baseline in the study population**

| **LTCs** | **N** | **Prevalence (%)** |
| --- | --- | --- |
| **Obesity** ^a^ | **54,483** | **20.76%** |
| **Hypertension** | **33,830** | **12.89%** |
| **Esophagus, stomach and duodenum diseases** | **20,407** | **7.78%** |
| **Solid neoplasms** | **19,873** | **7.57%** |
| **Other genitourinary diseases** | **18,975** | **7.23%** |
| **IHD** | **18,130** | **6.91%** |
| **Other** **musculoskeletal and joint diseases** | **17,763** | **6.77%** |
| **Degenerative joint diseases (e.g. OA)** | **17,758** | **6.77%** |
| **Colitis and related diseases** | **15,786** | **6.01%** |
| **Dyslipidemia** | **14,828** | **5.65%** |
| **Allergy** | **10,547** | **4.02%** |
| **Asthma** | **10,251** | **3.91%** |
| **Diabetes** | **8,781** | **3.35%** |
| **PN** | **8,473** | **3.23%** |
| **Cataract and other lens diseases** | **8,165** | **3.11%** |
| **Venous/lymphatic diseases** | **7,103** | **2.71%** |
| **Chronic pancreas, biliary tract and gallbladder diseases** | **7,019** | **2.67%** |
| **Inflammatory arthropathies** | **6,729** | **2.56%** |
| **Dorsopathies** | **6,339** | **2.42%** |
| **Anemia** | **6,232** | **2.37%** |
| **Thyroid diseases** | **6,189** | **2.36%** |
| **Ear, nose and throat diseases** | **6,153** | **2.34%** |
| **AF** | **5,645** | **2.15%** |
| **Other eye diseases** | **5,366** | **2.04%** |
| **Prostate diseases** | **5,096** | **1.94%** |
| **Other digestive diseases** | **3,914** | **1.49%** |
| **CVD** | **3,721** | **1.42%** |
| **COPD, CB and emphysema** | **3,276** | **1.25%** |
| **Depression and mood diseases** | **3,071** | **1.17%** |
| HF | 2,550 | 0.97% |
| Other cardiovascular diseases | 2,410 | 0.92% |
| Osteoporosis | 2,409 | 0.92% |
| Cardiac valve diseases | 2,405 | 0.92% |
| IBD | 2,312 | 0.88% |
| Other neurological diseases | 2,271 | 0.87% |
| Sleep disorders | 2,135 | 0.81% |
| Other metabolic diseases | 2,000 | 0.76% |
| Autoimmune diseases | 1,938 | 0.74% |
| PVD | 1,748 | 0.67% |
| CKD | 1,623 | 0.62% |
| Epilepsy | 1,453 | 0.55% |
| Neurotic, stress-related and somatoform diseases | 1,380 | 0.53% |
| Other psychiatric and behavioral diseases | 1,350 | 0.51% |
| Blood and blood forming organ diseases | 1,277 | 0.49% |
| Glaucoma | 1,275 | 0.49% |
| HN | 1,257 | 0.48% |
| Migraine and facial pain syndromes | 1,163 | 0.44% |
| Other respiratory diseases | 1,030 | 0.39% |
| Deafness and HI | 1,006 | 0.38% |
| Bradycardias and conduction diseases | 958 | 0.37% |
| CLD | 819 | 0.31% |
| Other skin diseases | 674 | 0.26% |
| MS | 586 | 0.22% |
| Chronic ulcer of the skin | 550 | 0.21% |
| Parkinson and parkinsonism | 348 | 0.13% |
| Schizophrenia and delusional diseases | 304 | 0.12% |
| Chronic infectious diseases | 274 | 0.10% |
| Blindness and VI | 271 | 0.10% |
| Chromosomal abnormalities | 25 | 0.01% |

Notes: ^a^ Obesity was defined as a BMI of ≥30 Kg/m^2^.

LTCs with a prevalence of ≥1% were included in the analyses (bold lines).

Abbreviations: UKB, UK Biobank; LTC, long-term condition; IHD, ischemic heart disease; E.g., for example; OA, osteoarthritis; PN, peripheral neuropathy; AF, atrial fibrillation; CVD, cerebrovascular disease; COPD, chronic obstructive pulmonary disease; CB, chronic bronchitis; HF, heart failure; IBD, inflammatory bowel disease; PVD, peripheral vascular disease; CKD, chronic kidney disease; HN, hematological neoplasm; HI, hearing impairment; CLD, chronic liver diseases; MS, multiple sclerosis; VI, visual impairment; BMI, body mass index.

**Supplementary Table 3: Information of LTCs at baseline in each cluster**

| **Cluster** | **N** | **LTC** | **Proportion within the pattern (%)** | **Observed/ expected ratio** | **Exclusivity (%)** |
| --- | --- | --- | --- | --- | --- |
| **Multimorbidity Pattern A**  (obesity accompanied with other disorders) | 46,172 | Allergy | 5.19% | 1.29 | 22.74% |
|  |  | Anemia | 2.64% | 1.11 | 19.58% |
|  |  | Asthma | 5.06% | 1.29 | 22.78% |
|  |  | AF | 3.06% | 1.42 | 25.05% |
|  |  | Cataract and other lens diseases | 3.90% | 1.25 | 22.06% |
|  |  | Chronic pancreas, biliary tract and gallbladder diseases | 4.65% | 1.74 | 30.57% |
|  |  | Colitis and related diseases | 7.55% | 1.26 | 22.08% |
|  |  | COPD, CB and emphysema | 1.55% | 1.24 | 21.83% |
|  |  | CVD | 1.94% | 1.37 | 24.05% |
|  |  | Degenerative joint diseases (e.g. OA) | 10.93% | 1.62 | 28.42% |
|  |  | Depression and mood diseases | 1.78% | 1.53 | 26.83% |
|  |  | Diabetes | 6.34% | 1.90 | 33.34% |
|  |  | Dorsopathies | 3.35% | 1.39 | 24.39% |
|  |  | Dyslipidemia | 7.18% | 1.27 | 22.36% |
|  |  | Ear, nose and throat diseases | 3.01% | 1.28 | 22.56% |
|  |  | Esophagus, stomach and duodenum diseases | 9.80% | 1.26 | 22.18% |
|  |  | Hypertension | 20.83% | 1.62 | 28.43% |
|  |  | Inflammatory arthropathies | 3.57% | 1.39 | 24.51% |
|  |  | IHD | 9.15% | 1.33 | 23.31% |
|  |  | Obesity | 100.00% | 4.82 | 84.75% |
|  |  | Other digestive diseases | 1.65% | 1.11 | 19.49% |
|  |  | Other eye diseases | 2.42% | 1.19 | 20.85% |
|  |  | Other genitourinary diseases | 8.73% | 1.21 | 21.23% |
|  |  | Other musculoskeletal and joint diseases | 9.04% | 1.34 | 23.51% |
|  |  | PN | 5.29% | 1.64 | 28.84% |
|  |  | Prostate diseases | 2.04% | 1.05 | 18.50% |
|  |  | Solid neoplasms | 8.10% | 1.07 | 18.81% |
|  |  | Thyroid diseases | 3.54% | 1.50 | 26.39% |
|  |  | Venous/lymphatic diseases | 3.42% | 1.27 | 22.26% |
| **Multimorbidity Pattern B** (cardio-cerebrovascular/respiratory/metabolic/musculoskeletal/depressive disorders) | 24,504 | Allergy | 13.97% | 3.48 | 32.46% |
|  |  | Anemia | 7.01% | 2.95 | 27.57% |
|  |  | Asthma | 13.72% | 3.51 | 32.79% |
|  |  | AF | 8.49% | 3.95 | 36.86% |
|  |  | Cataract and other lens diseases | 7.37% | 2.37 | 22.12% |
|  |  | Chronic pancreas, biliary tract and gallbladder diseases | 5.39% | 2.01 | 18.81% |
|  |  | Colitis and related diseases | 14.01% | 2.33 | 21.75% |
|  |  | COPD, CB and emphysema | 5.02% | 4.02 | 37.52% |
|  |  | CVD | 5.51% | 3.89 | 36.31% |
|  |  | Degenerative joint diseases (e.g. OA) | 18.78% | 2.78 | 25.91% |
|  |  | Depression and mood diseases | 3.23% | 2.76 | 25.76% |
|  |  | Diabetes | 15.62% | 4.67 | 43.58% |
|  |  | Dorsopathies | 6.71% | 2.78 | 25.95% |
|  |  | Dyslipidemia | 36.77% | 6.51 | 60.76% |
|  |  | Ear, nose and throat diseases | 3.82% | 1.63 | 15.21% |
|  |  | Esophagus, stomach and duodenum diseases | 18.18% | 2.34 | 21.83% |
|  |  | Hypertension | 91.05% | 7.06 | 65.95% |
|  |  | Inflammatory arthropathies | 9.37% | 3.65 | 34.11% |
|  |  | IHD | 37.72% | 5.46 | 50.99% |
|  |  | Obesity | 19.35% | 0.93 | 8.70% |
|  |  | Other digestive diseases | 3.55% | 2.38 | 22.25% |
|  |  | Other eye diseases | 4.67% | 2.29 | 21.34% |
|  |  | Other genitourinary diseases | 13.69% | 1.89 | 17.68% |
|  |  | Other musculoskeletal and joint diseases | 15.80% | 2.33 | 21.80% |
|  |  | PN | 6.99% | 2.16 | 20.21% |
|  |  | Prostate diseases | 4.91% | 2.53 | 23.59% |
|  |  | Solid neoplasms | 12.96% | 1.71 | 15.98% |
|  |  | Thyroid diseases | 7.19% | 3.05 | 28.49% |
|  |  | Venous/lymphatic diseases | 3.48% | 1.29 | 12.01% |
| **Multimorbidity Pattern C** (tumor/genitourinary/digestive disorders) | 67,147 | Allergy | 6.29% | 1.57 | 40.06% |
|  |  | Anemia | 4.33% | 1.83 | 46.69% |
|  |  | Asthma | 6.07% | 1.55 | 39.75% |
|  |  | AF | 2.90% | 1.35 | 34.49% |
|  |  | Cataract and other lens diseases | 5.95% | 1.91 | 48.89% |
|  |  | Chronic pancreas, biliary tract and gallbladder diseases | 4.58% | 1.71 | 43.77% |
|  |  | Colitis and related diseases | 12.03% | 2.00 | 51.18% |
|  |  | COPD, CB and emphysema | 1.86% | 1.49 | 38.16% |
|  |  | CVD | 1.86% | 1.31 | 33.62% |
|  |  | Degenerative joint diseases (e.g. OA) | 10.77% | 1.59 | 40.74% |
|  |  | Depression and mood diseases | 1.94% | 1.66 | 42.40% |
|  |  | Diabetes | 2.49% | 0.74 | 19.02% |
|  |  | Dorsopathies | 4.27% | 1.77 | 45.21% |
|  |  | Dyslipidemia | 2.61% | 0.46 | 11.80% |
|  |  | Ear, nose and throat diseases | 5.07% | 2.16 | 55.34% |
|  |  | Esophagus, stomach and duodenum diseases | 15.39% | 1.98 | 50.64% |
|  |  | Hypertension | 0.23% | 0.02 | 0.46% |
|  |  | Inflammatory arthropathies | 3.78% | 1.47 | 37.69% |
|  |  | IHD | 5.69% | 0.82 | 21.09% |
|  |  | Obesity | 0.00% | 0.00 | 0.00% |
|  |  | Other digestive diseases | 3.09% | 2.07 | 53.07% |
|  |  | Other eye diseases | 4.08% | 1.99 | 51.01% |
|  |  | Other genitourinary diseases | 15.34% | 2.12 | 54.27% |
|  |  | Other musculoskeletal and joint diseases | 12.94% | 1.91 | 48.91% |
|  |  | PN | 5.75% | 1.78 | 45.58% |
|  |  | Prostate diseases | 4.00% | 2.06 | 52.73% |
|  |  | Solid neoplasms | 17.29% | 2.28 | 58.43% |
|  |  | Thyroid diseases | 3.63% | 1.54 | 39.38% |
|  |  | Venous/lymphatic diseases | 6.10% | 2.26 | 57.71% |

Abbreviations: LTC, long-term condition; AF, atrial fibrillation; COPD, chronic obstructive pulmonary disease; CB, chronic bronchitis; CVD, cerebrovascular disease; E.g., for example; OA, osteoarthritis; IHD, ischemic heart disease; PN, peripheral neuropathy.

**Supplementary Table 4: LTCs that have the potential to characterize each** **cluster**

| **Cluster** | **N** | **LTC** | **Proportion within the pattern (%)** | **Observed/ expected ratio** | **Exclusivity (%)** |
| --- | --- | --- | --- | --- | --- |
| **Multimorbidity Pattern A**  (obesity accompanied with other disorders) | 46,172 | Obesity (BMI≥30 Kg/m2) | 100.00% | 4.82 | 84.75% |
|  |  | Hypertension | 20.83% | 1.62 | 28.43% |
|  |  | Degenerative joint diseases (e.g. OA) | 10.93% | 1.62 | 28.42% |
|  |  | Diabetes | 6.34% | 1.90 | 33.34% |
|  |  | PN | 5.29% | 1.64 | 28.84% |
|  |  | Chronic pancreas, biliary tract and gallbladder diseases | 4.65% | 1.74 | 30.57% |
|  |  | Thyroid diseases | 3.54% | 1.50 | 26.39% |
|  |  | AF | 3.06% | 1.42 | 25.05% |
|  |  | Depression and mood diseases | 1.78% | 1.53 | 26.83% |
| **Multimorbidity Pattern B** (cardio-cerebrovascular/respiratory/metabolic/musculoskeletal/depressive disorders) | 24,504 | Hypertension | 91.05% | 7.06 | 65.95% |
|  |  | IHD | 37.72% | 5.46 | 50.99% |
|  |  | Dyslipidemia | 36.77% | 6.51 | 60.76% |
|  |  | Degenerative joint diseases (e.g. OA) | 18.78% | 2.78 | 25.91% |
|  |  | Diabetes | 15.62% | 4.67 | 43.58% |
|  |  | Allergy | 13.97% | 3.48 | 32.46% |
|  |  | Asthma | 13.72% | 3.51 | 32.79% |
|  |  | Inflammatory arthropathies | 9.37% | 3.65 | 34.11% |
|  |  | AF | 8.49% | 3.95 | 36.86% |
|  |  | Thyroid diseases | 7.19% | 3.05 | 28.49% |
|  |  | Anemia | 7.01% | 2.95 | 27.57% |
|  |  | Dorsopathies | 6.71% | 2.78 | 25.95% |
|  |  | CVD | 5.51% | 3.89 | 36.31% |
|  |  | COPD, CB and emphysema | 5.02% | 4.02 | 37.52% |
|  |  | Depression and mood diseases | 3.23% | 2.76 | 25.76% |
|  |  | Esophagus, stomach and duodenum diseases | 18.18% | 2.34 | 21.83% |
|  |  | Other musculoskeletal and joint diseases | 15.80% | 2.33 | 21.80% |
|  |  | Colitis and related diseases | 14.01% | 2.33 | 21.75% |
|  |  | Cataract and other lens diseases | 7.37% | 2.37 | 22.12% |
|  |  | PN | 6.99% | 2.16 | 20.21% |
|  |  | Chronic pancreas, biliary tract and gallbladder diseases | 5.39% | 2.01 | 18.81% |
|  |  | Prostate diseases | 4.91% | 2.53 | 23.59% |
|  |  | Other eye diseases | 4.67% | 2.29 | 21.34% |
|  |  | Other digestive diseases | 3.55% | 2.38 | 22.25% |
| **Multimorbidity Pattern C** (tumor/genitourinary/digestive disorders) | 67,147 | Solid neoplasms | 17.29% | 2.28 | 58.43% |
|  |  | Other genitourinary diseases | 15.34% | 2.12 | 54.27% |
|  |  | Colitis and related diseases | 12.03% | 2.00 | 51.18% |
|  |  | Venous/lymphatic diseases | 6.10% | 2.26 | 57.71% |
|  |  | Ear, nose and throat diseases | 5.07% | 2.16 | 55.34% |
|  |  | Prostate diseases | 4.00% | 2.06 | 52.73% |
|  |  | Other digestive diseases | 3.09% | 2.07 | 53.07% |
|  |  | Esophagus, stomach and duodenum diseases | 15.39% | 1.98 | 50.64% |
|  |  | Other musculoskeletal and joint diseases | 12.94% | 1.91 | 48.91% |
|  |  | Degenerative joint diseases (e.g. OA) | 10.77% | 1.59 | 40.74% |
|  |  | Allergy | 6.29% | 1.57 | 40.06% |
|  |  | Asthma | 6.07% | 1.55 | 39.75% |
|  |  | Cataract and other lens diseases | 5.95% | 1.91 | 48.89% |
|  |  | PN | 5.75% | 1.78 | 45.58% |
|  |  | Chronic pancreas, biliary tract and gallbladder diseases | 4.58% | 1.71 | 43.77% |
|  |  | Anemia | 4.33% | 1.83 | 46.69% |
|  |  | Dorsopathies | 4.27% | 1.77 | 45.21% |
|  |  | Other eye diseases | 4.08% | 1.99 | 51.01% |
|  |  | Inflammatory arthropathies | 3.78% | 1.47 | 37.69% |
|  |  | Thyroid diseases | 3.63% | 1.54 | 39.38% |
|  |  | AF | 2.90% | 1.35 | 34.49% |
|  |  | Depression and mood diseases | 1.94% | 1.66 | 42.40% |
|  |  | CVD | 1.86% | 1.31 | 33.62% |
|  |  | COPD, CB and emphysema | 1.86% | 1.49 | 38.16% |

LTCs have the potential to characterize each cluster if their exclusivity was≥25% OR observed/expected ratio ≥2 .

LTCs with both exclusivity ≥25% AND observed/expected ratio ≥2 were considered as characterizing each cluster (gray bands).

Abbreviations: LTC, long-term condition; E.g., for example; OA, osteoarthritis; PN, peripheral neuropathy; AF, atrial fibrillation; IHD, ischemic heart disease; CVD, cerebrovascular disease; COPD, chronic obstructive pulmonary disease; CB, chronic bronchitis.

**Supplementary Table 5: The list of LTCs** **included in each pattern**

| **Cluster** | **N** | **LTC** | **Proportion within the pattern (%)** | **Observed/ expected ratio** | **Exclusivity (%)** |
| --- | --- | --- | --- | --- | --- |
| **Multimorbidity Pattern A**  (obesity accompanied with other disorders) | 46,172 | Obesity (BMI≥30 Kg/m2) | 100.00% | 4.82 | 84.75% |
| **Multimorbidity Pattern B** (cardio-cerebrovascular/respiratory/metabolic/musculoskeletal/depressive disorders) | 24,504 | Hypertension | 91.05% | 7.06 | 65.95% |
|  |  | IHD | 37.72% | 5.46 | 50.99% |
|  |  | Dyslipidemia | 36.77% | 6.51 | 60.76% |
|  |  | Degenerative joint diseases (e.g. OA) | 18.78% | 2.78 | 25.91% |
|  |  | Diabetes | 15.62% | 4.67 | 43.58% |
|  |  | Allergy | 13.97% | 3.48 | 32.46% |
|  |  | Asthma | 13.72% | 3.51 | 32.79% |
|  |  | Inflammatory arthropathies | 9.37% | 3.65 | 34.11% |
|  |  | AF | 8.49% | 3.95 | 36.86% |
|  |  | Thyroid diseases | 7.19% | 3.05 | 28.49% |
|  |  | Anemia | 7.01% | 2.95 | 27.57% |
|  |  | Dorsopathies | 6.71% | 2.78 | 25.95% |
|  |  | CVD | 5.51% | 3.89 | 36.31% |
|  |  | COPD, CB and emphysema | 5.02% | 4.02 | 37.52% |
|  |  | Depression and mood diseases | 3.23% | 2.76 | 25.76% |
| **Multimorbidity Pattern C** (tumor/genitourinary/digestive disorders) | 67,147 | Solid neoplasms | 17.29% | 2.28 | 58.43% |
|  |  | Other genitourinary diseases | 15.34% | 2.12 | 54.27% |
|  |  | Colitis and related diseases | 12.03% | 2.00 | 51.18% |
|  |  | Venous/lymphatic diseases | 6.10% | 2.26 | 57.71% |
|  |  | Ear, nose and throat diseases | 5.07% | 2.16 | 55.34% |
|  |  | Prostate diseases | 4.00% | 2.06 | 52.73% |
|  |  | Other digestive diseases | 3.09% | 2.07 | 53.07% |

LTCs with both exclusivity ≥25% and observed/expected ratio ≥2 were included in each pattern.

Abbreviations: LTC, long-term condition; E.g., for example; IHD, ischemic heart disease; OA, osteoarthritis; AF, atrial fibrillation; CVD, cerebrovascular disease; COPD, chronic obstructive pulmonary disease; CB, chronic bronchitis.

**Supplementary Table 6: Participant characteristics across eight categories according to the multimorbidity status**

| **Variables** | **Healthy**  **(0 LTC)** |  | **LTC Counts** | | | |  | **Multimorbidity Patterns** | | |
| --- | --- | --- | --- | --- | --- | --- | --- | --- | --- | --- |
|  |  |  | **1 LTC** | **2** **LTCs** | **3 LTCs** | **4 or more LTCs** |  | **Pattern A** | **Pattern B** | **Pattern C** |
| **Participants, n (%)** | 107,660 |  | 54,153 | 34,741 | 20,299 | 28,630 |  | 46,172 | 24,504 | 67,147 |
| **Age, mean (SD), y** | 61.89 (4.05) |  | 62.24 (4.06) | 62.62 (4.08) | 62.90 (4.04) | 63.31 (4.00) |  | 62.19 (4.08) | 63.63 (3.88) | 62.62 (4.07) |
| **Female, n (%)** | 56,898 (52.85) |  | 30,238 (55.84) | 18,621 (53.60) | 10,431 (51.39) | 14,309 (49.98) |  | 24,811 (53.74) | 10,842 (44.25) | 37,946 (56.51) |
| **Higher Education** ^a^**, where reported, n (%)** | 50,076 (59.73) |  | 22,913 (57.46) | 13,455 (55.35) | 7,407 (54.05) | 9,154 (52.36) |  | 16,160 (52.42) | 8,421 (53.49) | 28,348 (58.09) |
| **BMI, where reported, mean (SD), kg/m^2^** | 26.47 (3.92) |  | 27.40 (4.47) | 28.91 (4.90) | 29.49 (5.12) | 30.88 (5.46) |  | 33.89 (3.76) | 28.01 (4.50) | 25.57 (2.66) |
| **Physical Activity, where reported, mean (SD), MET-min/week** | 2753.74 (2713.13) |  | 2756.21 (2723.24) | 2677.80 (2730.35) | 2620.42 (2791.12) | 2311.27 (2633.46) |  | 2348.35 (2625.54) | 2527.35 (2709.94) | 2845.08 (2769.63) |
| **Smoking Status, where reported, n (%)** |  | | | | | | | | | |
| Current | 9,713 |  | 5,122 | 3,185 | 1,934 | 2,725 |  | 3,836 | 2,361 | 6,769 |
| Previous | 40,978 |  | 21,330 | 14,776 | 8,929 | 13,615 |  | 21,060 | 11,322 | 26,268 |
| Never | 56,387 |  | 27,371 | 16,538 | 9,254 | 11,986 |  | 20,922 | 10,562 | 33,665 |
| ***APOE4* Carriers, where reported, n (%)** | 27,204 (28.67) |  | 13,496 (28.27) | 8,667 (28.41) | 5,051 (28.43) | 6,954 (28.33) |  | 11,203 (27.77) | 5,995 (28.57) | 16,970 (28.66) |
| **Incident Dementia During Follow-up , n (%)** |  | | | | | | | | | |
| ACD | 1,750 (1.63) |  | 977 (1.80) | 773 (2.23) | 528 (2.60) | 1,095 (3.82) |  | 967 (2.09) | 959 (3.91) | 1,447 (2.15) |
| AD | 827 (0.77) |  | 433 (0.80) | 350 (1.01) | 211 (1.04) | 407 (1.42) |  | 359 (0.78) | 385 (1.57) | 657 (0.98) |
| VD | 314 (0.29) |  | 219 (0.40) | 181 (0.52) | 141 (0.69) | 379 (1.32) |  | 284 (0.62) | 304 (1.24) | 332 (0.49) |

Notes: ^a^ Higher education refers to “with college/university degree or other professional qualification”.

Data presented as mean (SD) for continuous variables and number (%) for categorical variables.

Among incident dementia cases, ACD, AD, and VD were treated as separate outcomes.

Abbreviations: UKB, UK Biobank; ACD, all-cause dementia; AD, Alzheimer’s disease, VD, vascular dementia; SD, standard deviation; BMI, body mass index; MET, metabolic equivalent; *APOE4*, *apolipoprotein E4*; LTC, long-term condition.

**Supplementary Table 7: Risks of dementia in participants with individual LTCs at baseline**

| **LTC** | **ACD HR (95% CI)** | **P Value** | **AD  HR (95% CI)** | **P Value** | **VD  HR (95% CI)** | **P Value** |
| --- | --- | --- | --- | --- | --- | --- |
| AF | 1.6 (1.29-1.99) | **2.63E-05** | 1.3 (0.9-1.89) | 0.168 | 3.75 (2.56-5.5) | **1.38E-11** |
| Allergy | 1.21 (0.97-1.53) | 0.094 | 1.16 (0.82-1.65) | 0.404 | 1.88 (1.2-2.96) | **0.006** |
| Anemia | 1.39 (1.05-1.84) | **0.021** | 1.08 (0.67-1.73) | 0.764 | 3.48 (2.21-5.48) | **8.08E-08** |
| Asthma | 1.23 (0.98-1.55) | 0.08 | 1.17 (0.82-1.67) | 0.395 | 1.95 (1.24-3.06) | **0.004** |
| Cataract and other lens diseases | 1.22 (0.97-1.54) | 0.094 | 1.23 (0.87-1.75) | 0.243 | 1.77 (1.11-2.83) | **0.017** |
| Chronic pancreas, biliary tract and gallbladder diseases | 1.13 (0.84-1.53) | 0.417 | 0.79 (0.47-1.34) | 0.382 | 2.13 (1.24-3.64) | **0.006** |
| Colitis and related diseases | 1.32 (1.11-1.57) | **0.002** | 1.09 (0.83-1.45) | 0.531 | 1.74 (1.19-2.54) | **0.004** |
| COPD, CB and emphysema | 1.68 (1.19-2.36) | **0.003** | 1.26 (0.69-2.31) | 0.453 | 3.74 (2.16-6.49) | **2.69E-06** |
| CVD | 2.47 (1.92-3.16) | **9.37E-13** | 0.85 (0.45-1.6) | 0.612 | 8.38 (5.84-12.01) | **6.74E-31** |
| Degenerative joint diseases (e.g. OA) | 1.44 (1.22-1.69) | **1.80E-05** | 1.48 (1.16-1.89) | **0.002** | 1.78 (1.24-2.56) | **0.002** |
| Depression and mood diseases | 3.83 (2.97-4.94) | **4.44E-25** | 2.66 (1.68-4.19) | **2.71E-05** | 6.67 (4.14-10.75) | **5.83E-15** |
| Diabetes | 2.4 (1.98-2.9) | **2.22E-19** | 2.29 (1.68-3.11) | **1.28E-07** | 5.37 (3.81-7.57) | **9.22E-22** |
| Dorsopathies | 1.97 (1.57-2.48) | **5.23E-09** | 1.75 (1.21-2.52) | **0.003** | 3.26 (2.1-5.05) | **1.26E-07** |
| Dyslipidemia | 1.5 (1.27-1.78) | **2.34E-06** | 1.37 (1.04-1.79) | **0.023** | 3.09 (2.25-4.24) | **3.48E-12** |
| Ear, nose and throat diseases | 1.24 (0.94-1.65) | 0.133 | 1.16 (0.75-1.81) | 0.501 | 1.82 (1.03-3.24) | **0.04** |
| Esophagus, stomach and duodenum diseases | 1.2 (1.01-1.42) | **0.037** | 1.06 (0.81-1.38) | 0.674 | 2.09 (1.51-2.9) | **1.08E-05** |
| Hypertension | 1.41 (1.24-1.61) | **3.17E-07** | 1.24 (1.01-1.52) | **0.045** | 2.39 (1.82-3.14) | **3.70E-10** |
| IHD | 1.5 (1.29-1.75) | **1.20E-07** | 1.39 (1.09-1.77) | **0.008** | 2.94 (2.19-3.95) | **8.05E-13** |
| Inflammatory arthropathies | 1.69 (1.34-2.13) | **1.05E-05** | 1.34 (0.91-1.98) | 0.142 | 2.91 (1.88-4.5) | **1.67E-06** |
| Obesity | 1.1 (0.93-1.29) | 0.259 | 1.15 (0.89-1.49) | 0.297 | 1.41 (1-1.98) | **0.05** |
| Other digestive diseases | 1.09 (0.76-1.57) | 0.626 | 0.78 (0.42-1.47) | 0.441 | 1.4 (0.65-3) | 0.392 |
| Other eye diseases | 1.11 (0.83-1.49) | 0.483 | 1.38 (0.93-2.04) | 0.108 | 0.93 (0.43-1.99) | 0.845 |
| Other genitourinary diseases | 1.3 (1.09-1.54) | **0.003** | 1.2 (0.92-1.55) | 0.171 | 1.96 (1.38-2.78) | **1.90E-04** |
| Other musculoskeletal and joint diseases | 1.27 (1.07-1.51) | **0.006** | 1.25 (0.97-1.62) | 0.09 | 1.59 (1.09-2.32) | **0.017** |
| PN | 1.49 (1.17-1.89) | **0.001** | 1.25 (0.85-1.84) | 0.262 | 2.38 (1.49-3.79) | **2.61E-04** |
| Prostate diseases | 1.03 (0.79-1.33) | 0.835 | 1.21 (0.83-1.77) | 0.323 | 1.13 (0.61-2.08) | 0.701 |
| Solid neoplasms | 1.01 (0.85-1.2) | 0.9 | 1.03 (0.8-1.33) | 0.826 | 1.25 (0.85-1.84) | 0.252 |
| Thyroid diseases | 1.73 (1.32-2.25) | **5.35E-05** | 1.45 (0.95-2.19) | 0.081 | 2.68 (1.6-4.5) | **1.91E-04** |
| Venous/lymphatic diseases | 1.13 (0.84-1.52) | 0.408 | 1.45 (0.99-2.13) | 0.059 | 1.67 (0.92-3.04) | 0.089 |

HRs and 95% CIs for dementia according to baseline LTC were calculated using Cox proportional hazard regression with healthy participants as reference after adjusting for age, sex, education, BMI, physical activity, smoking and *APOE4* status.

Abbreviations: ACD, all-cause dementia; AD, Alzheimer's disease; VD, vascular dementia; LTC, long-term condition; IHD, ischemic heart disease; E.g., for example; OA, osteoarthritis; CVD, cerebrovascular disease; AF, atrial fibrillation; COPD, chronic obstructive pulmonary disease; CB, chronic bronchitis; PN, peripheral neuropathy; BMI, body mass index; *APOE4, apolipoprotein E4*.

**Supplementary Table 8: Risks of dementia in males with** **individual LTCs at baseline**

| **LTC** | **ACD HR (95% CI)** | **P Value** | **AD  HR (95% CI)** | **P Value** | **VD  HR (95% CI)** | **P Value** |
| --- | --- | --- | --- | --- | --- | --- |
| AF | 1.61 (1.26-2.06) | **1.37E-04** | 1.42 (0.94-2.15) | 0.097 | 4.39 (2.88-6.7) | **5.98E-12** |
| Allergy | 1.01 (0.72-1.4) | 0.975 | 0.99 (0.59-1.68) | 0.979 | 1.84 (1-3.4) | 0.051 |
| Anemia | 1.35 (0.94-1.96) | 0.108 | 0.91 (0.45-1.85) | 0.794 | 4.3 (2.5-7.38) | **1.25E-07** |
| Asthma | 0.99 (0.71-1.39) | 0.972 | 0.96 (0.56-1.66) | 0.892 | 1.91 (1.03-3.53) | **0.039** |
| Degenerative joint diseases (e.g. OA) | 1.11 (0.81-1.52) | 0.51 | 1.4 (0.89-2.2) | 0.146 | 1.47 (0.76-2.86) | 0.256 |
| Depression and mood diseases | 1.09 (0.69-1.72) | 0.725 | 0.44 (0.14-1.37) | 0.155 | 2.08 (0.9-4.8) | 0.086 |
| Diabetes | 1.17 (0.92-1.48) | 0.215 | 0.81 (0.51-1.27) | 0.351 | 1.9 (1.17-3.09) | **0.01** |
| Dorsopathies | 1.73 (1.13-2.65) | **0.011** | 1.01 (0.41-2.48) | 0.979 | 4.8 (2.56-9.01) | **1.05E-06** |
| Dyslipidemia | 2.36 (1.76-3.17) | **1.11E-08** | 0.95 (0.47-1.94) | 0.892 | 8.25 (5.34-12.72) | **1.50E-21** |
| Cataract and other lens diseases | 1.28 (1.03-1.6) | **0.026** | 1.35 (0.96-1.91) | 0.085 | 1.69 (1.05-2.72) | **0.032** |
| Chronic pancreas, biliary tract and gallbladder diseases | 3.79 (2.71-5.29) | **7.10E-15** | 2.07 (1.02-4.23) | **0.045** | 7.58 (4.14-13.86) | **4.86E-11** |
| Colitis and related diseases | 2.12 (1.68-2.67) | **1.80E-10** | 1.92 (1.29-2.87) | **0.001** | 5.93 (3.99-8.82) | **1.30E-18** |
| COPD, CB and emphysema | 1.59 (1.14-2.21) | **0.006** | 1.67 (1-2.79) | 0.051 | 2.3 (1.14-4.62) | **0.019** |
| CVD | 1.4 (1.14-1.71) | **0.001** | 1.25 (0.9-1.75) | 0.189 | 3.45 (2.38-5) | **5.55E-11** |
| Ear, nose and throat diseases | 1.26 (0.91-1.76) | 0.169 | 1.17 (0.68-2.02) | 0.564 | 2.47 (1.34-4.55) | **0.004** |
| Esophagus, stomach and duodenum diseases | 0.98 (0.78-1.24) | 0.876 | 0.8 (0.54-1.2) | 0.283 | 2.09 (1.37-3.18) | **6.10E-04** |
| Hypertension | 1.25 (1.06-1.48) | **0.008** | 1.16 (0.88-1.52) | 0.287 | 2.77 (1.98-3.86) | **2.00E-09** |
| IHD | 1.37 (1.14-1.63) | **5.59E-04** | 1.17 (0.87-1.58) | 0.307 | 3.32 (2.37-4.65) | **3.52E-12** |
| Inflammatory arthropathies | 1.94 (1.46-2.58) | **5.87E-06** | 1.74 (1.08-2.81) | **0.024** | 4.39 (2.67-7.22) | **5.08E-09** |
| Obesity | 1.04 (0.85-1.28) | 0.7 | 1.04 (0.74-1.46) | 0.811 | 1.35 (0.88-2.06) | 0.167 |
| Other digestive diseases | 1.01 (0.61-1.66) | 0.974 | 0.47 (0.15-1.47) | 0.195 | 1.83 (0.74-4.53) | 0.193 |
| Other eye diseases | 0.99 (0.66-1.46) | 0.941 | 0.96 (0.51-1.81) | 0.898 | 1.11 (0.45-2.76) | 0.818 |
| Other genitourinary diseases | 1.22 (0.95-1.56) | 0.123 | 1.37 (0.95-1.99) | 0.095 | 1.96 (1.19-3.21) | **0.008** |
| Other musculoskeletal and joint diseases | 1.2 (0.96-1.49) | 0.108 | 1.26 (0.9-1.77) | 0.173 | 1.7 (1.06-2.71) | **0.026** |
| PN | 1.51 (1.09-2.09) | **0.014** | 1.46 (0.86-2.48) | 0.163 | 2.58 (1.39-4.8) | **0.003** |
| Prostate diseases | 1.03 (0.79-1.34) | 0.812 | 1.22 (0.83-1.79) | 0.305 | 1.16 (0.63-2.14) | 0.637 |
| Solid neoplasms | 0.86 (0.68-1.09) | 0.224 | 0.9 (0.62-1.3) | 0.559 | 1.25 (0.75-2.07) | 0.392 |
| Thyroid diseases | 1.15 (0.65-2.04) | 0.637 | 0.74 (0.24-2.32) | 0.604 | 2.75 (1.1-6.84) | **0.03** |
| Venous/lymphatic diseases | 0.87 (0.54-1.39) | 0.548 | 0.84 (0.39-1.78) | 0.648 | 2.01 (0.93-4.38) | 0.077 |

HRs and 95% CIs for dementia according to baseline LTC were calculated using Cox proportional hazard regression with healthy participants as reference after adjusting for age, education, BMI, physical activity, smoking and *APOE4* status.

Abbreviations: ACD, all-cause dementia; AD, Alzheimer's disease; VD, vascular dementia; LTC, long-term condition; IHD, ischemic heart disease; E.g., for example; OA, osteoarthritis; CVD, cerebrovascular disease; AF, atrial fibrillation; COPD, chronic obstructive pulmonary disease; CB, chronic bronchitis; PN, peripheral neuropathy; BMI, body mass index; *APOE4, apolipoprotein E4*.

**Supplementary Table 9: Risks of dementia in females with individual LTCs at baseline**

| **LTC** | **ACD HR (95% CI)** | **P Value** | **AD  HR (95% CI)** | **P Value** | **VD  HR (95% CI)** | **P Value** |
| --- | --- | --- | --- | --- | --- | --- |
| AF | 1.55 (0.95-2.54) | 0.08 | 0.88 (0.36-2.15) | 0.779 | 2.06 (0.74-5.79) | 0.169 |
| Allergy | 1.48 (1.07-2.05) | **0.017** | 1.36 (0.85-2.18) | 0.205 | 1.87 (0.94-3.7) | 0.072 |
| Anemia | 1.46 (0.95-2.26) | 0.086 | 1.3 (0.69-2.47) | 0.419 | 2.25 (0.95-5.3) | 0.064 |
| Asthma | 1.53 (1.11-2.11) | **0.01** | 1.41 (0.88-2.26) | 0.159 | 1.93 (0.98-3.82) | 0.058 |
| Cataract and other lens diseases | 1.4 (0.99-2) | 0.058 | 1.05 (0.61-1.83) | 0.85 | 2.15 (1.1-4.2) | **0.024** |
| Chronic pancreas, biliary tract and gallbladder diseases | 1.17 (0.79-1.74) | 0.432 | 1.02 (0.56-1.85) | 0.945 | 2.03 (1-4.14) | 0.051 |
| Colitis and related diseases | 1.53 (1.19-1.98) | **0.001** | 1.41 (0.98-2.04) | 0.064 | 1.51 (0.82-2.76) | 0.185 |
| COPD, CB and emphysema | 1.61 (0.9-2.89) | 0.109 | 1.57 (0.69-3.58) | 0.278 | 2.05 (0.62-6.74) | 0.237 |
| CVD | 2.89 (1.83-4.57) | **5.17E-06** | 0.59 (0.15-2.39) | 0.46 | 9.24 (4.85-17.61) | **1.45E-11** |
| Degenerative joint diseases (e.g. OA) | 1.69 (1.32-2.18) | **4.19E-05** | 1.64 (1.15-2.34) | **0.006** | 1.94 (1.11-3.4) | **0.02** |
| Depression and mood diseases | 3.88 (2.62-5.75) | **1.20E-11** | 3.37 (1.85-6.12) | **6.73E-05** | 5.5 (2.51-12.07) | **2.14E-05** |
| Diabetes | 3.37 (2.41-4.71) | **1.22E-12** | 3.3 (2.05-5.32) | **9.16E-07** | 4.06 (1.99-8.3) | **1.18E-04** |
| Dorsopathies | 2.53 (1.84-3.48) | **1.15E-08** | 1.85 (1.1-3.1) | **0.021** | 4.42 (2.46-7.91) | **6.00E-07** |
| Dyslipidemia | 1.86 (1.37-2.54) | **7.62E-05** | 1.68 (1.07-2.63) | **0.024** | 2.39 (1.28-4.48) | **0.006** |
| Ear, nose and throat diseases | 1.21 (0.71-2.07) | 0.488 | 1.15 (0.54-2.46) | 0.715 | 0.47 (0.06-3.42) | 0.457 |
| Esophagus, stomach and duodenum diseases | 1.56 (1.21-2) | **4.89E-04** | 1.4 (0.98-2) | 0.067 | 2.09 (1.23-3.53) | **0.006** |
| Hypertension | 1.74 (1.4-2.15) | **3.97E-07** | 1.36 (0.99-1.87) | 0.06 | 1.78 (1.1-2.89) | **0.019** |
| IHD | 2.05 (1.55-2.72) | **5.09E-07** | 2.04 (1.38-3.03) | **3.68E-04** | 2.08 (1.11-3.89) | **0.022** |
| Inflammatory arthropathies | 1.34 (0.9-2.02) | 0.152 | 0.9 (0.46-1.76) | 0.753 | 1.02 (0.36-2.87) | 0.97 |
| Obesity | 1.23 (0.94-1.62) | 0.127 | 1.33 (0.88-2) | 0.173 | 1.5 (0.84-2.66) | 0.167 |
| Other digestive diseases | 1.24 (0.74-2.09) | 0.411 | 1.12 (0.52-2.38) | 0.775 | 0.9 (0.22-3.74) | 0.889 |
| Other eye diseases | 1.3 (0.84-2.01) | 0.232 | 1.85 (1.12-3.06) | **0.017** | 0.65 (0.16-2.68) | 0.549 |
| Other genitourinary diseases | 1.39 (1.09-1.76) | **0.007** | 1.09 (0.76-1.56) | 0.654 | 1.98 (1.2-3.27) | **0.008** |
| Other musculoskeletal and joint diseases | 1.42 (1.07-1.88) | **0.014** | 1.23 (0.82-1.85) | 0.314 | 1.41 (0.74-2.7) | 0.298 |
| PN | 1.49 (1.04-2.13) | **0.029** | 1.11 (0.62-1.96) | 0.729 | 2.17 (1.07-4.39) | **0.031** |
| Prostate diseases | - | - | - | - | - | - |
| Solid neoplasms | 1.24 (0.96-1.6) | 0.093 | 1.19 (0.84-1.69) | 0.337 | 1.28 (0.71-2.33) | 0.412 |
| Thyroid diseases | 1.97 (1.45-2.67) | **1.51E-05** | 1.68 (1.06-2.65) | **0.027** | 2.51 (1.33-4.75) | **0.005** |
| Venous/lymphatic diseases | 1.43 (0.97-2.09) | 0.069 | 1.97 (1.25-3.1) | **0.004** | 1.35 (0.53-3.4) | 0.527 |

HRs and 95% CIs for dementia according to baseline LTC were calculated using Cox proportional hazard regression with healthy participants as reference after adjusting for age, education, BMI, physical activity, smoking and *APOE4* status.

Abbreviations: ACD, all-cause dementia; AD, Alzheimer's disease; VD, vascular dementia; LTC, long-term condition; IHD, ischemic heart disease; E.g., for example; OA, osteoarthritis; CVD, cerebrovascular disease; AF, atrial fibrillation; COPD, chronic obstructive pulmonary disease; CB, chronic bronchitis; PN, peripheral neuropathy; BMI, body mass index; *APOE4, apolipoprotein E4.*

**Supplementary Table 10: Risks of dementia in the mid-aged with individual LTCs at baseline**

| **LTC** | **ACD HR (95% CI)** | **P Value** | **AD  HR (95% CI)** | **P Value** | **VD  HR (95% CI)** | **P Value** |
| --- | --- | --- | --- | --- | --- | --- |
| AF | 1.93 (1.35-2.77) | **3.28E-04** | 1.82 (1.04-3.17) | **0.035** | 6.66 (3.68-12.03) | **3.35E-10** |
| Allergy | 1.11 (0.76-1.61) | 0.589 | 0.77 (0.4-1.47) | 0.428 | 1.24 (0.48-3.15) | 0.658 |
| Anemia | 1.42 (0.91-2.2) | 0.122 | 0.88 (0.39-1.98) | 0.753 | 3.81 (1.79-8.11) | **5.05E-04** |
| Asthma | 1.15 (0.8-1.68) | 0.451 | 0.8 (0.42-1.52) | 0.498 | 1.28 (0.5-3.27) | 0.604 |
| Cataract and other lens diseases | 0.88 (0.53-1.46) | 0.63 | 0.72 (0.32-1.63) | 0.431 | 0.77 (0.19-3.19) | 0.721 |
| Chronic pancreas, biliary tract and gallbladder diseases | 1.18 (0.74-1.87) | 0.482 | 0.9 (0.42-1.95) | 0.797 | 1.53 (0.54-4.39) | 0.426 |
| Colitis and related diseases | 1.11 (0.82-1.51) | 0.487 | 0.45 (0.23-0.88) | **0.019** | 2.24 (1.22-4.1) | **0.009** |
| COPD, CB and emphysema | 1.83 (1.04-3.21) | **0.035** | 0.96 (0.3-3.03) | 0.943 | 6.1 (2.67-13.95) | **1.84E-05** |
| CVD | 3.18 (2.18-4.64) | **2.16E-09** | 1.18 (0.48-2.89) | 0.716 | 13.31 (7.58-23.36) | **2.01E-19** |
| Degenerative joint diseases (e.g. OA) | 1.37 (1.04-1.8) | **0.024** | 1.33 (0.88-1.99) | 0.175 | 1.82 (0.98-3.41) | 0.06 |
| Depression and mood diseases | 3.66 (2.53-5.28) | **5.55E-12** | 1.76 (0.82-3.78) | 0.146 | 7.5 (3.71-15.18) | **2.08E-08** |
| Diabetes | 3.03 (2.26-4.04) | **7.85E-14** | 2.29 (1.39-3.76) | **0.001** | 8.79 (5.18-14.93) | **8.33E-16** |
| Dorsopathies | 1.59 (1.09-2.34) | **0.017** | 1.22 (0.64-2.32) | 0.551 | 2.81 (1.3-6.04) | **0.008** |
| Dyslipidemia | 1.65 (1.26-2.17) | **3.01E-04** | 1.27 (0.81-2) | 0.299 | 4.03 (2.39-6.79) | **1.76E-07** |
| Ear, nose and throat diseases | 1.33 (0.87-2.01) | 0.184 | 1.14 (0.58-2.23) | 0.709 | 2.18 (0.93-5.14) | 0.074 |
| Esophagus, stomach and duodenum diseases | 1.17 (0.89-1.53) | 0.254 | 0.98 (0.64-1.5) | 0.931 | 2.33 (1.35-4.02) | **0.002** |
| Hypertension | 1.53 (1.24-1.9) | **8.08E-05** | 1.39 (1.01-1.92) | **0.045** | 3.05 (1.94-4.79) | **1.45E-06** |
| IHD | 1.69 (1.32-2.17) | **3.21E-05** | 1.35 (0.9-2.02) | 0.153 | 4.19 (2.59-6.77) | **4.75E-09** |
| Inflammatory arthropathies | 1.75 (1.2-2.55) | **0.003** | 1.13 (0.57-2.22) | 0.731 | 3.23 (1.54-6.78) | **0.002** |
| Obesity | 1.08 (0.85-1.38) | 0.539 | 1.07 (0.73-1.58) | 0.731 | 1.52 (0.89-2.59) | 0.126 |
| Other digestive diseases | 1.04 (0.57-1.9) | 0.89 | 0.41 (0.1-1.64) | 0.207 | 1.94 (0.6-6.27) | 0.27 |
| Other eye diseases | 0.89 (0.52-1.52) | 0.674 | 0.68 (0.28-1.67) | 0.402 | 0.9 (0.22-3.72) | 0.883 |
| Other genitourinary diseases | 1.3 (0.99-1.71) | 0.062 | 0.97 (0.62-1.53) | 0.91 | 2.12 (1.17-3.86) | **0.014** |
| Other musculoskeletal and joint diseases | 1.41 (1.09-1.83) | **0.009** | 1.26 (0.85-1.89) | 0.249 | 1.68 (0.9-3.14) | 0.104 |
| PN | 1.27 (0.87-1.86) | 0.21 | 0.56 (0.25-1.28) | 0.169 | 2.61 (1.28-5.3) | **0.008** |
| Prostate diseases | 1.27 (0.81-1.99) | 0.296 | 0.85 (0.37-1.93) | 0.691 | 2.08 (0.8-5.42) | 0.133 |
| Solid neoplasms | 0.96 (0.71-1.29) | 0.768 | 0.89 (0.57-1.39) | 0.615 | 1.24 (0.61-2.55) | 0.549 |
| Thyroid diseases | 1.46 (0.94-2.27) | 0.095 | 1.09 (0.53-2.25) | 0.814 | 2.76 (1.13-6.76) | **0.026** |
| Venous/lymphatic diseases | 0.94 (0.57-1.53) | 0.794 | 1.49 (0.84-2.63) | 0.169 | 0.74 (0.18-3.07) | 0.681 |

HRs and 95% CIs for dementia according to baseline LTC were calculated using Cox proportional hazard regression with healthy participants as reference after adjusting for age, sex, education, BMI, physical activity, smoking and *APOE4* status.

Abbreviations: ACD, all-cause dementia; AD, Alzheimer's disease; VD, vascular dementia; LTC, long-term condition; IHD, ischemic heart disease; E.g., for example; OA, osteoarthritis; CVD, cerebrovascular disease; AF, atrial fibrillation; COPD, chronic obstructive pulmonary disease; CB, chronic bronchitis; PN, peripheral neuropathy; BMI, body mass index; *APOE4, apolipoprotein E4*.

**Supplementary Table 11: Risks of dementia in the old-aged with individual LTCs at baseline**

| **LTC** | **ACD HR (95% CI)** | **P Value** | **AD  HR (95% CI)** | **P Value** | **VD  HR (95% CI)** | **P Value** |
| --- | --- | --- | --- | --- | --- | --- |
| AF | 1.46 (1.11-1.92) | **0.008** | 1.06 (0.64-1.75) | 0.826 | 2.75 (1.66-4.54) | **7.79E-05** |
| Allergy | 1.27 (0.95-1.7) | 0.102 | 1.45 (0.95-2.21) | 0.083 | 2.17 (1.29-3.68) | **0.004** |
| Anemia | 1.39 (0.96-2) | 0.08 | 1.24 (0.69-2.21) | 0.478 | 3.31 (1.87-5.85) | **4.01E-05** |
| Asthma | 1.26 (0.94-1.7) | 0.118 | 1.44 (0.94-2.22) | 0.093 | 2.24 (1.33-3.79) | **0.003** |
| Cataract and other lens diseases | 1.39 (1.06-1.81) | **0.016** | 1.49 (1.01-2.21) | **0.046** | 2.09 (1.26-3.46) | **0.004** |
| Chronic pancreas, biliary tract and gallbladder diseases | 1.1 (0.74-1.63) | 0.642 | 0.72 (0.35-1.47) | 0.362 | 2.43 (1.3-4.54) | **0.006** |
| Colitis and related diseases | 1.46 (1.17-1.8) | **6.18E-04** | 1.55 (1.13-2.13) | **0.007** | 1.5 (0.92-2.45) | 0.101 |
| COPD, CB and emphysema | 1.63 (1.06-2.51) | **0.027** | 1.48 (0.72-3.01) | 0.285 | 2.84 (1.35-5.96) | **0.006** |
| CVD | 2.14 (1.54-2.96) | **5.45E-06** | 0.67 (0.27-1.63) | 0.375 | 6.45 (4.03-10.31) | **7.01E-15** |
| Degenerative joint diseases (e.g. OA) | 1.49 (1.21-1.84) | **1.49E-04** | 1.6 (1.18-2.18) | **0.003** | 1.79 (1.15-2.8) | **0.01** |
| Depression and mood diseases | 3.96 (2.79-5.63) | **1.76E-14** | 3.61 (2.04-6.4) | **1.09E-05** | 6.05 (3.15-11.6) | **6.02E-08** |
| Diabetes | 2.05 (1.59-2.64) | **2.50E-08** | 2.31 (1.56-3.41) | **2.61E-05** | 3.91 (2.48-6.17) | **4.49E-09** |
| Dorsopathies | 2.26 (1.7-3) | **1.86E-08** | 2.19 (1.4-3.41) | **5.79E-04** | 3.56 (2.08-6.07) | **3.35E-06** |
| Dyslipidemia | 1.43 (1.15-1.77) | **0.001** | 1.44 (1.03-2.01) | **0.035** | 2.7 (1.82-4.02) | **9.60E-07** |
| Ear, nose and throat diseases | 1.16 (0.79-1.7) | 0.45 | 1.16 (0.65-2.09) | 0.616 | 1.55 (0.71-3.37) | 0.27 |
| Esophagus, stomach and duodenum diseases | 1.22 (0.98-1.51) | 0.078 | 1.12 (0.8-1.57) | 0.519 | 1.97 (1.31-2.98) | **0.001** |
| Hypertension | 1.34 (1.13-1.59) | **6.05E-04** | 1.14 (0.87-1.5) | 0.326 | 2.1 (1.5-2.94) | **1.73E-05** |
| IHD | 1.42 (1.17-1.71) | **3.24E-04** | 1.43 (1.06-1.93) | **0.019** | 2.43 (1.68-3.52) | **2.62E-06** |
| Inflammatory arthropathies | 1.65 (1.23-2.23) | **9.25E-04** | 1.48 (0.92-2.38) | 0.108 | 2.75 (1.6-4.73) | **2.43E-04** |
| Obesity | 1.12 (0.9-1.39) | 0.321 | 1.21 (0.86-1.72) | 0.279 | 1.34 (0.86-2.09) | 0.192 |
| Other digestive diseases | 1.13 (0.72-1.76) | 0.604 | 1.03 (0.51-2.09) | 0.936 | 1.15 (0.42-3.15) | 0.787 |
| Other eye diseases | 1.24 (0.87-1.76) | 0.234 | 1.8 (1.15-2.8) | **0.01** | 0.93 (0.38-2.31) | 0.879 |
| Other genitourinary diseases | 1.29 (1.04-1.6) | **0.023** | 1.34 (0.98-1.85) | 0.069 | 1.87 (1.21-2.89) | **0.005** |
| Other musculoskeletal and joint diseases | 1.18 (0.94-1.49) | 0.155 | 1.24 (0.88-1.75) | 0.219 | 1.55 (0.96-2.49) | 0.07 |
| PN | 1.65 (1.21-2.25) | **0.002** | 1.87 (1.19-2.93) | **0.006** | 2.19 (1.18-4.08) | **0.013** |
| Prostate diseases | 0.93 (0.68-1.28) | 0.661 | 1.39 (0.9-2.14) | 0.142 | 0.83 (0.37-1.83) | 0.641 |
| Solid neoplasms | 1.05 (0.85-1.3) | 0.663 | 1.12 (0.82-1.53) | 0.485 | 1.27 (0.8-2) | 0.313 |
| Thyroid diseases | 1.92 (1.38-2.68) | **1.08E-04** | 1.74 (1.04-2.9) | **0.034** | 2.67 (1.41-5.05) | **0.002** |
| Venous/lymphatic diseases | 1.27 (0.88-1.84) | 0.209 | 1.42 (0.84-2.41) | 0.192 | 2.21 (1.14-4.28) | **0.019** |

HRs and 95% CIs for dementia according to baseline LTC were calculated using Cox proportional hazard regression with healthy participants as reference after adjusting for age, sex, education, BMI, physical activity, smoking and *APOE4* status.

Abbreviations: ACD, all-cause dementia; AD, Alzheimer's disease; VD, vascular dementia; LTC, long-term condition; IHD, ischemic heart disease; E.g., for example; OA, osteoarthritis; CVD, cerebrovascular disease; AF, atrial fibrillation; COPD, chronic obstructive pulmonary disease; CB, chronic bronchitis; PN, peripheral neuropathy; BMI, body mass index; *APOE4, apolipoprotein E4*.

**Supplementary Table 12: Risks of dementia in *APOE4* non-carriers with individual LTCs at baseline**

| **LTC** | **ACD HR (95% CI)** | **P Value** | **AD  HR (95% CI)** | **P Value** | **VD  HR (95% CI)** | **P Value** |
| --- | --- | --- | --- | --- | --- | --- |
| AF | 1.73 (1.28-2.34) | **3.77E-04** | 1.37 (0.76-2.44) | 0.293 | 3 (1.69-5.31) | **1.68E-04** |
| Allergy | 1.1 (0.78-1.55) | 0.573 | 0.75 (0.38-1.48) | 0.411 | 1.91 (1.02-3.6) | **0.045** |
| Anemia | 1.09 (0.69-1.73) | 0.712 | 0.63 (0.23-1.69) | 0.356 | 2.72 (1.34-5.51) | **0.006** |
| Asthma | 1.11 (0.79-1.58) | 0.541 | 0.78 (0.39-1.54) | 0.471 | 1.98 (1.05-3.74) | **0.035** |
| Cataract and other lens diseases | 1.23 (0.88-1.72) | 0.234 | 1.66 (1.01-2.74) | **0.047** | 1.75 (0.91-3.37) | 0.096 |
| Chronic pancreas, biliary tract and gallbladder diseases | 1.13 (0.72-1.77) | 0.593 | 0.85 (0.37-1.95) | 0.701 | 1.89 (0.85-4.24) | 0.12 |
| Colitis and related diseases | 1.45 (1.14-1.84) | **0.003** | 1.05 (0.66-1.66) | 0.836 | 2.21 (1.35-3.61) | **0.002** |
| COPD, CB and emphysema | 1.47 (0.87-2.48) | 0.147 | 0.28 (0.04-2.02) | 0.207 | 3.69 (1.71-7.93) | **8.40E-04** |
| CVD | 3.17 (2.29-4.39) | **3.24E-12** | 0.9 (0.33-2.45) | 0.841 | 10.5 (6.52-16.91) | **3.80E-22** |
| Degenerative joint diseases (e.g. OA) | 1.34 (1.05-1.71) | **0.021** | 1.28 (0.85-1.95) | 0.241 | 1.67 (1-2.79) | 0.052 |
| Depression and mood diseases | 4.44 (3.13-6.3) | **6.01E-17** | 2.71 (1.32-5.58) | **0.007** | 7.65 (4.12-14.23) | **1.25E-10** |
| Diabetes | 2.5 (1.92-3.25) | **1.25E-11** | 2.67 (1.7-4.21) | **2.19E-05** | 4.57 (2.81-7.43) | **8.64E-10** |
| Dorsopathies | 1.72 (1.21-2.46) | **0.003** | 1.37 (0.72-2.62) | 0.34 | 2.39 (1.17-4.87) | **0.017** |
| Dyslipidemia | 1.66 (1.3-2.11) | **4.02E-05** | 1.41 (0.91-2.19) | 0.122 | 3.35 (2.15-5.21) | **8.16E-08** |
| Ear, nose and throat diseases | 1.13 (0.73-1.74) | 0.588 | 0.99 (0.46-2.12) | 0.979 | 2.03 (0.92-4.45) | 0.078 |
| Esophagus, stomach and duodenum diseases | 1.1 (0.85-1.41) | 0.464 | 0.93 (0.59-1.45) | 0.744 | 2.11 (1.33-3.36) | **0.002** |
| Hypertension | 1.49 (1.24-1.8) | **3.26E-05** | 1.4 (1.01-1.93) | **0.043** | 2.41 (1.65-3.54) | **6.16E-06** |
| IHD | 1.68 (1.35-2.08) | **2.38E-06** | 1.68 (1.16-2.45) | **0.006** | 3.48 (2.33-5.21) | **1.28E-09** |
| Inflammatory arthropathies | 1.95 (1.41-2.69) | **4.69E-05** | 1.51 (0.83-2.75) | 0.182 | 3.37 (1.9-6.01) | **3.57E-05** |
| Obesity | 1.14 (0.9-1.44) | 0.273 | 1.09 (0.72-1.65) | 0.689 | 1.4 (0.88-2.21) | 0.156 |
| Other digestive diseases | 0.96 (0.55-1.67) | 0.889 | 0.2 (0.03-1.4) | 0.105 | 0.8 (0.2-3.29) | 0.759 |
| Other eye diseases | 0.8 (0.49-1.33) | 0.396 | 0.7 (0.29-1.71) | 0.433 | 1.34 (0.53-3.34) | 0.537 |
| Other genitourinary diseases | 1.21 (0.93-1.57) | 0.15 | 0.87 (0.54-1.42) | 0.577 | 1.48 (0.84-2.6) | 0.173 |
| Other musculoskeletal and joint diseases | 1.18 (0.92-1.52) | 0.195 | 1.13 (0.74-1.73) | 0.576 | 1.49 (0.87-2.52) | 0.143 |
| PN | 1.36 (0.94-1.98) | 0.107 | 1.32 (0.7-2.46) | 0.391 | 2.16 (1.08-4.33) | **0.029** |
| Prostate diseases | 1.07 (0.74-1.55) | 0.706 | 1.25 (0.68-2.31) | 0.472 | 1.38 (0.65-2.96) | 0.402 |
| Solid neoplasms | 0.92 (0.71-1.19) | 0.52 | 0.75 (0.47-1.2) | 0.233 | 1.14 (0.64-2.02) | 0.652 |
| Thyroid diseases | 2.21 (1.56-3.13) | **7.90E-06** | 1.85 (1.02-3.33) | **0.042** | 3.84 (2.02-7.29) | **4.00E-05** |
| Venous/lymphatic diseases | 0.94 (0.59-1.52) | 0.809 | 1.22 (0.62-2.4) | 0.563 | 1.77 (0.76-4.12) | 0.184 |

HRs and 95% CIs for dementia according to baseline LTC were calculated using Cox proportional hazard regression with healthy participants as reference after adjusting for age, sex, education, BMI, physical activity and smoking.

Abbreviations: *APOE4, apolipoprotein E4;* ACD, all-cause dementia; AD, Alzheimer's disease; VD, vascular dementia; LTC, long-term condition; IHD, ischemic heart disease; E.g., for example; OA, osteoarthritis; CVD, cerebrovascular disease; AF, atrial fibrillation; COPD, chronic obstructive pulmonary disease; CB, chronic bronchitis; PN, peripheral neuropathy; BMI, body mass index.

**Supplementary Table 13: Risks of dementia in *APOE4* carriers with individual LTCs at baseline**

| **LTC** | **ACD HR (95% CI)** | **P Value** | **AD  HR (95% CI)** | **P Value** | **VD  HR (95% CI)** | **P Value** |
| --- | --- | --- | --- | --- | --- | --- |
| AF | 1.48 (1.07-2.03) | **0.017** | 1.26 (0.78-2.05) | 0.35 | 4.6 (2.74-7.72) | **7.63E-09** |
| Allergy | 1.32 (0.97-1.79) | 0.073 | 1.43 (0.95-2.16) | 0.087 | 1.86 (0.97-3.58) | 0.061 |
| Anemia | 1.67 (1.17-2.37) | **0.005** | 1.35 (0.79-2.33) | 0.271 | 4.23 (2.33-7.68) | **2.10E-06** |
| Asthma | 1.34 (0.98-1.82) | 0.064 | 1.42 (0.94-2.16) | 0.098 | 1.93 (1-3.7) | **0.049** |
| Cataract and other lens diseases | 1.22 (0.88-1.68) | 0.234 | 0.97 (0.59-1.59) | 0.902 | 1.78 (0.91-3.49) | 0.091 |
| Chronic pancreas, biliary tract and gallbladder diseases | 1.14 (0.76-1.7) | 0.529 | 0.76 (0.39-1.48) | 0.416 | 2.39 (1.16-4.92) | **0.018** |
| Colitis and related diseases | 1.2 (0.93-1.54) | 0.167 | 1.13 (0.79-1.61) | 0.514 | 1.26 (0.68-2.34) | 0.469 |
| COPD, CB and emphysema | 1.89 (1.2-2.98) | **0.006** | 1.94 (1.02-3.69) | **0.043** | 3.78 (1.7-8.38) | **0.001** |
| CVD | 1.86 (1.26-2.74) | **0.002** | 0.82 (0.36-1.85) | 0.63 | 6.45 (3.68-11.29) | **6.94E-11** |
| Degenerative joint diseases (e.g. OA) | 1.54 (1.23-1.92) | **1.60E-04** | 1.61 (1.19-2.19) | **0.002** | 1.92 (1.15-3.21) | **0.012** |
| Depression and mood diseases | 3.32 (2.29-4.82) | **2.60E-10** | 2.63 (1.46-4.74) | **0.001** | 5.78 (2.71-12.3) | **5.39E-06** |
| Diabetes | 2.31 (1.75-3.04) | **2.42E-09** | 2.03 (1.33-3.08) | **9.21E-04** | 6.38 (3.93-10.35) | **6.57E-14** |
| Dorsopathies | 2.22 (1.65-2.99) | **1.41E-07** | 2 (1.29-3.12) | **0.002** | 4.23 (2.42-7.41) | **4.42E-07** |
| Dyslipidemia | 1.39 (1.09-1.76) | **0.007** | 1.35 (0.96-1.9) | 0.087 | 2.9 (1.83-4.58) | **5.30E-06** |
| Ear, nose and throat diseases | 1.35 (0.93-1.96) | 0.114 | 1.27 (0.74-2.19) | 0.383 | 1.65 (0.71-3.84) | 0.241 |
| Esophagus, stomach and duodenum diseases | 1.29 (1.03-1.62) | **0.028** | 1.15 (0.83-1.6) | 0.404 | 2.08 (1.31-3.32) | **0.002** |
| Hypertension | 1.34 (1.12-1.61) | **0.002** | 1.14 (0.87-1.5) | 0.343 | 2.4 (1.62-3.54) | **1.12E-05** |
| IHD | 1.36 (1.1-1.69) | **0.004** | 1.22 (0.89-1.68) | 0.219 | 2.45 (1.58-3.79) | **6.42E-05** |
| Inflammatory arthropathies | 1.47 (1.04-2.06) | **0.028** | 1.25 (0.75-2.08) | 0.401 | 2.41 (1.22-4.75) | **0.011** |
| Obesity | 1.07 (0.85-1.34) | 0.588 | 1.19 (0.86-1.67) | 0.293 | 1.45 (0.87-2.41) | 0.152 |
| Other digestive diseases | 1.26 (0.78-2.02) | 0.343 | 1.2 (0.62-2.34) | 0.591 | 2.09 (0.84-5.21) | 0.114 |
| Other eye diseases | 1.38 (0.96-1.98) | 0.079 | 1.79 (1.16-2.78) | **0.009** | 0.54 (0.13-2.19) | 0.385 |
| Other genitourinary diseases | 1.37 (1.09-1.72) | **0.006** | 1.4 (1.03-1.9) | **0.033** | 2.41 (1.53-3.79) | **1.51E-04** |
| Other musculoskeletal and joint diseases | 1.36 (1.07-1.72) | **0.01** | 1.34 (0.96-1.85) | 0.083 | 1.69 (0.98-2.91) | 0.057 |
| PN | 1.61 (1.17-2.2) | **0.003** | 1.22 (0.74-2.01) | 0.428 | 2.63 (1.4-4.94) | **0.003** |
| Prostate diseases | 0.98 (0.68-1.42) | 0.91 | 1.18 (0.73-1.92) | 0.499 | 0.81 (0.29-2.28) | 0.687 |
| Solid neoplasms | 1.1 (0.87-1.38) | 0.416 | 1.21 (0.89-1.63) | 0.226 | 1.39 (0.82-2.34) | 0.22 |
| Thyroid diseases | 1.31 (0.87-1.98) | 0.197 | 1.18 (0.65-2.14) | 0.578 | 1.53 (0.6-3.88) | 0.373 |
| Venous/lymphatic diseases | 1.31 (0.9-1.91) | 0.165 | 1.61 (1-2.57) | **0.048** | 1.61 (0.7-3.74) | 0.264 |

HRs and 95% CIs for dementia according to baseline LTC were calculated using Cox proportional hazard regression with healthy participants as reference after adjusting for age, sex, education, BMI, physical activity and smoking.

Abbreviations: *APOE4, apolipoprotein E4;* ACD, all-cause dementia; AD, Alzheimer's disease; VD, vascular dementia; LTC, long-term condition; IHD, ischemic heart disease; E.g., for example; OA, osteoarthritis; CVD, cerebrovascular disease; AF, atrial fibrillation; COPD, chronic obstructive pulmonary disease; CB, chronic bronchitis; PN, peripheral neuropathy; BMI, body mass index.

**Supplementary Table 14: Subgroup analyses for the association between multimorbidity status and dementia by sex**

| **Variables** | **ACD** | | | |  | **AD** | | | |  | **VD** | | | |
| --- | --- | --- | --- | --- | --- | --- | --- | --- | --- | --- | --- | --- | --- | --- |
|  | **Participants** | **Events** | **HR (95%CI)** | **P Value** |  | **Participants** | **Events** | **HR (95%CI)** | **P Value** |  | **Participants** | **Events** | **HR (95%CI)** | **P Value** |
| **Males** |  |  |  |  |  |  |  |  |  |  |  |  |  |  |
| 0 LTC/healthy | 31,230 | 504 | Reference | - |  | 31,230 | 205 | Reference | - |  | 31,230 | 80 | Reference | - |
| 1 LTC | 13,667 | 235 | 0.96 (0.82-1.12) | 0.614 |  | 13,667 | 88 | 0.89 (0.69-1.15) | 0.369 |  | 13,667 | 64 | 1.63 (1.17-2.27) | **0.004** |
| 2 LTCs | 8,599 | 173 | 1.04 (0.87-1.24) | 0.649 |  | 8,599 | 79 | 1.17 (0.9-1.52) | 0.241 |  | 8,599 | 45 | 1.68 (1.16-2.43) | **0.006** |
| 3 LTCs | 5,092 | 111 | 1.07 (0.87-1.32) | 0.53 |  | 5,092 | 46 | 1.11 (0.8-1.53) | 0.542 |  | 5,092 | 36 | 2.15 (1.44-3.2) | **1.74E-04** |
| 4 or more LTCs | 6,352 | 211 | 1.55 (1.31-1.83) | **3.61E-07** |  | 6,352 | 64 | 1.2 (0.9-1.61) | 0.218 |  | 6,352 | 79 | 3.59 (2.59-4.97) | **1.66E-14** |
| Multimorbidity Pattern A | 10,760 | 197 | 1.11 (0.9-1.35) | 0.329 |  | 10,760 | 72 | 1.09 (0.78-1.51) | 0.617 |  | 10,760 | 54 | 1.72 (1.14-2.6) | **0.01** |
| Multimorbidity Pattern B | 6,524 | 188 | 1.3 (1.09-1.54) | **0.003** |  | 6,524 | 71 | 1.23 (0.94-1.62) | 0.136 |  | 6,524 | 68 | 2.8 (2.02-3.89) | **7.20E-10** |
| Multimorbidity Pattern C | 16,426 | 345 | 1.03 (0.9-1.19) | 0.639 |  | 16,426 | 134 | 0.98 (0.78-1.21) | 0.824 |  | 16,426 | 102 | 1.91 (1.42-2.57) | **1.70E-05** |
| **Females** |  |  |  |  |  |  |  |  |  |  |  |  |  |  |
| 0 LTC/healthy | 30,399 | 300 | Reference | - |  | 30,399 | 163 | Reference | - |  | 30,399 | 50 | Reference | - |
| 1 LTC | 15,337 | 188 | 1.13 (0.94-1.36) | 0.184 |  | 15,337 | 101 | 1.14 (0.89-1.46) | 0.306 |  | 15,337 | 25 | 0.89 (0.55-1.43) | 0.62 |
| 2 LTCs | 8,610 | 135 | 1.35 (1.1-1.66) | **0.004** |  | 8,610 | 60 | 1.16 (0.86-1.57) | 0.322 |  | 8,610 | 29 | 1.65 (1.04-2.62) | **0.035** |
| 3 LTCs | 4,546 | 79 | 1.39 (1.08-1.79) | **0.01** |  | 4,546 | 36 | 1.23 (0.86-1.78) | 0.26 |  | 4,546 | 21 | 2.06 (1.22-3.46) | **0.006** |
| 4 or more LTCs | 5,378 | 135 | 1.86 (1.5-2.3) | **1.46E-08** |  | 5,378 | 56 | 1.56 (1.13-2.14) | **0.006** |  | 5,378 | 35 | 2.56 (1.62-4.06) | **5.74E-05** |
| Multimorbidity Pattern A | 10,594 | 138 | 1.22 (0.95-1.57) | 0.118 |  | 10,594 | 60 | 1.26 (0.86-1.83) | 0.231 |  | 10,594 | 32 | 1.31 (0.76-2.26) | 0.331 |
| Multimorbidity Pattern B | 4,349 | 119 | 1.78 (1.43-2.2) | **1.72E-07** |  | 4,349 | 47 | 1.35 (0.97-1.87) | 0.073 |  | 4,349 | 24 | 1.96 (1.19-3.21) | **0.008** |
| Multimorbidity Pattern C | 18,928 | 280 | 1.25 (1.06-1.47) | **0.007** |  | 18,928 | 146 | 1.18 (0.94-1.48) | 0.145 |  | 18,928 | 54 | 1.46 (0.99-2.15) | 0.055 |

HRs and 95% CIs for dementia according to multimorbidity status were calculated using Cox proportional hazard regression with healthy participants as reference after adjusting for age, education, BMI, physical activity, smoking and *APOE4* status.

Abbreviations: ACD, all-cause dementia; AD, Alzheimer’s disease; VD, vascular dementia; LTC, long-term condition; BMI, body mass index; *APOE4*, *apolipoprotein E4*.

**Supplementary Table 15: Subgroup analyses for the association between multimorbidity status and dementia by age**

| **Variables** | **ACD** | | | |  | **AD** | | | |  | **VD** | | | |
| --- | --- | --- | --- | --- | --- | --- | --- | --- | --- | --- | --- | --- | --- | --- |
|  | **Participants** | **Events** | **HR (95%CI)** | **P Value** |  | **Participants** | **Events** | **HR (95%CI)** | **P Value** |  | **Participants** | **Events** | **HR (95%CI)** | **P Value** |
| **The mid-aged** |  |  |  |  |  |  |  |  |  |  |  |  |  |  |
| 0 LTC/healthy | 45,626 | 357 | Reference | - |  | 45,626 | 169 | Reference | - |  | 45,626 | 49 | Reference | - |
| 1 LTC | 20,620 | 182 | 1.03 (0.86-1.24) | 0.726 |  | 20,620 | 80 | 0.95 (0.73-1.25) | 0.725 |  | 20,620 | 30 | 1.24 (0.79-1.96) | 0.354 |
| 2 LTCs | 11,609 | 121 | 1.17 (0.95-1.44) | 0.151 |  | 11,609 | 52 | 1.06 (0.77-1.45) | 0.736 |  | 11,609 | 26 | 1.8 (1.11-2.91) | **0.017** |
| 3 LTCs | 6,245 | 78 | 1.32 (1.03-1.69) | **0.029** |  | 6,245 | 33 | 1.2 (0.82-1.76) | 0.341 |  | 6,245 | 22 | 2.61 (1.56-4.36) | **2.45E-04** |
| 4 or more LTCs | 7,115 | 123 | 1.71 (1.38-2.12) | **1.04E-06** |  | 7,115 | 33 | 1.01 (0.69-1.49) | 0.943 |  | 7,115 | 44 | 4.22 (2.75-6.5) | **5.53E-11** |
| Multimorbidity Pattern A | 15,452 | 150 | 1.1 (0.87-1.39) | 0.431 |  | 15,452 | 56 | 1 (0.69-1.45) | 0.997 |  | 15,452 | 37 | 1.67 (1-2.79) | 0.051 |
| Multimorbidity Pattern B | 6,228 | 109 | 1.63 (1.31-2.02) | **1.07E-05** |  | 6,228 | 43 | 1.4 (1-1.96) | 0.052 |  | 6,228 | 33 | 3.3 (2.11-5.17) | **1.60E-07** |
| Multimorbidity Pattern C | 23,909 | 245 | 1.13 (0.96-1.33) | 0.136 |  | 23,909 | 99 | 0.93 (0.73-1.2) | 0.577 |  | 23,909 | 52 | 1.8 (1.21-2.67) | **0.003** |
| **The old-aged** |  |  |  |  |  |  |  |  |  |  |  |  |  |  |
| 0 LTC/healthy | 16,003 | 447 | Reference | - |  | 16,003 | 199 | Reference | - |  | 16,003 | 81 | Reference | - |
| 1 LTC | 8,384 | 241 | 1.02 (0.87-1.19) | 0.819 |  | 8,384 | 109 | 1.04 (0.83-1.32) | 0.715 |  | 8,384 | 59 | 1.37 (0.98-1.91) | 0.068 |
| 2 LTCs | 5,600 | 187 | 1.14 (0.96-1.35) | 0.14 |  | 5,600 | 87 | 1.23 (0.96-1.59) | 0.104 |  | 5,600 | 48 | 1.58 (1.1-2.26) | **0.013** |
| 3 LTCs | 3,393 | 112 | 1.1 (0.89-1.35) | 0.378 |  | 3,393 | 49 | 1.14 (0.83-1.56) | 0.414 |  | 3,393 | 35 | 1.86 (1.24-2.78) | **0.002** |
| 4 or more LTCs | 4,615 | 223 | 1.62 (1.37-1.92) | **1.91E-08** |  | 4,615 | 87 | 1.55 (1.19-2.02) | **0.001** |  | 4,615 | 70 | 2.72 (1.95-3.81) | **4.33E-09** |
| Multimorbidity Pattern A | 5,902 | 185 | 1.18 (0.95-1.45) | 0.132 |  | 5,902 | 76 | 1.3 (0.94-1.8) | 0.118 |  | 5,902 | 49 | 1.48 (0.96-2.27) | 0.074 |
| Multimorbidity Pattern B | 4,645 | 198 | 1.38 (1.16-1.63) | **2.08E-04** |  | 4,645 | 75 | 1.24 (0.95-1.62) | 0.114 |  | 4,645 | 59 | 2.17 (1.55-3.05) | **7.47E-06** |
| Multimorbidity Pattern C | 11,445 | 380 | 1.1 (0.96-1.26) | 0.169 |  | 11,445 | 181 | 1.16 (0.95-1.43) | 0.139 |  | 11,445 | 104 | 1.68 (1.25-2.24) | **5.18E-04** |

HRs and 95% CIs for dementia according to multimorbidity status were calculated using Cox proportional hazard regression with healthy participants as reference after adjusting for age, sex, education, BMI, physical activity, smoking and *APOE4* status.

Abbreviations: ACD, all-cause dementia; AD, Alzheimer’s disease; VD, vascular dementia; LTC, long-term condition; BMI, body mass index; *APOE4*, *apolipoprotein E4*.

**Supplementary Table 1****6: Subgroup analyses for the association between multimorbidity status and dementia by *APOE4* status**

| **Variables** | **ACD** | | | |  | **AD** | | | |  | **VD** | | | |
| --- | --- | --- | --- | --- | --- | --- | --- | --- | --- | --- | --- | --- | --- | --- |
|  | **Participants** | **Events** | **HR (95%CI)** | **P Value** |  | **Participants** | **Events** | **HR (95%CI)** | **P Value** |  | **Participants** | **Events** | **HR (95%CI)** | **P Value** |
| ***APOE4* Non-carriers** |  |  |  |  |  |  |  |  |  |  |  |  |  |  |
| 0 LTC/healthy | 43,898 | 368 | Reference | - |  | 43,898 | 136 | Reference | - |  | 43,898 | 62 | Reference | - |
| 1 LTC | 20,890 | 196 | 1 (0.84-1.19) | 0.982 |  | 20,890 | 66 | 0.9 (0.67-1.21) | 0.496 |  | 20,890 | 49 | 1.46 (1-2.13) | **0.048** |
| 2 LTCs | 12,355 | 142 | 1.13 (0.93-1.38) | 0.217 |  | 12,355 | 59 | 1.28 (0.94-1.75) | 0.118 |  | 12,355 | 34 | 1.53 (1.01-2.34) | **0.047** |
| 3 LTCs | 6,875 | 83 | 1.1 (0.86-1.4) | 0.444 |  | 6,875 | 29 | 1.06 (0.7-1.58) | 0.796 |  | 6,875 | 28 | 2.05 (1.31-3.23) | **0.002** |
| 4 or more LTCs | 8,414 | 171 | 1.65 (1.36-2) | **2.80E-07** |  | 8,414 | 42 | 1.14 (0.8-1.63) | 0.476 |  | 8,414 | 61 | 3.13 (2.16-4.54) | **1.95E-09** |
| Multimorbidity Pattern A | 15,472 | 167 | 1.1 (0.88-1.37) | 0.424 |  | 15,472 | 51 | 1.01 (0.68-1.49) | 0.975 |  | 15,472 | 45 | 1.31 (0.84-2.07) | 0.236 |
| Multimorbidity Pattern B | 7,781 | 160 | 1.58 (1.31-1.91) | **2.15E-06** |  | 7,781 | 52 | 1.42 (1.03-1.97) | **0.034** |  | 7,781 | 51 | 2.66 (1.82-3.87) | **3.68E-07** |
| Multimorbidity Pattern C | 25,281 | 265 | 1.04 (0.89-1.22) | 0.606 |  | 25,281 | 93 | 0.96 (0.73-1.25) | 0.753 |  | 25,281 | 76 | 1.83 (1.31-2.57) | **4.66E-04** |
| ***APOE4* Carriers** |  |  |  |  |  |  |  |  |  |  |  |  |  |  |
| 0 LTC/healthy | 17,731 | 436 | Reference | - |  | 17,731 | 232 | Reference | - |  | 17,731 | 68 | Reference | - |
| 1 LTC | 8,114 | 227 | 1.05 (0.89-1.23) | 0.554 |  | 8,114 | 123 | 1.07 (0.86-1.33) | 0.548 |  | 8,114 | 40 | 1.19 (0.81-1.76) | 0.383 |
| 2 LTCs | 4,854 | 166 | 1.17 (0.98-1.4) | 0.084 |  | 4,854 | 80 | 1.09 (0.84-1.41) | 0.514 |  | 4,854 | 40 | 1.81 (1.22-2.69) | **0.003** |
| 3 LTCs | 2,763 | 107 | 1.26 (1.02-1.56) | **0.034** |  | 2,763 | 53 | 1.22 (0.9-1.66) | 0.19 |  | 2,763 | 29 | 2.2 (1.42-3.43) | **4.52E-04** |
| 4 or more LTCs | 3,316 | 175 | 1.66 (1.38-1.99) | **6.88E-08** |  | 3,316 | 78 | 1.49 (1.14-1.94) | **0.004** |  | 3,316 | 53 | 3.25 (2.23-4.75) | **9.37E-10** |
| Multimorbidity Pattern A | 5,882 | 168 | 1.18 (0.95-1.47) | 0.135 |  | 5,882 | 81 | 1.27 (0.92-1.73) | 0.144 |  | 5,882 | 41 | 1.9 (1.17-3.08) | **0.009** |
| Multimorbidity Pattern B | 3,092 | 147 | 1.35 (1.12-1.63) | **0.002** |  | 3,092 | 66 | 1.19 (0.9-1.57) | 0.214 |  | 3,092 | 41 | 2.36 (1.59-3.49) | **1.89E-05** |
| Multimorbidity Pattern C | 10,073 | 360 | 1.18 (1.03-1.36) | **0.02** |  | 10,073 | 187 | 1.13 (0.93-1.38) | 0.203 |  | 10,073 | 80 | 1.66 (1.2-2.3) | **0.002** |

HRs and 95% CIs for dementia according to multimorbidity status were calculated using Cox proportional hazard regression with healthy participants as reference after adjusting for age, sex, education, BMI, physical activity and smoking.

Abbreviations: *APOE4*, *apolipoprotein E4;* ACD, all-cause dementia; AD, Alzheimer’s disease; VD, vascular dementia; LTC, long-term condition; BMI, body mass index.
